# New one-pot synthesis of 4-arylpyrazolo[3,4-*b*]pyridin-6-ones based on 5-aminopyrazoles and azlactones

**DOI:** 10.3762/bjoc.19.83

**Published:** 2023-08-02

**Authors:** Vladislav Yu Shuvalov, Ekaterina Yu Vlasova, Tatyana Yu Zheleznova, Alexander S Fisyuk

**Affiliations:** 1 Laboratory of New Organic Materials, Omsk State Technical University, 11 Mira Ave., 644050 Omsk, Russian Federation; 2 Department of Organic and Analytical Chemistry, F. M. Dostoevsky Omsk State University, Mira Ave., 55a, 644077 Omsk, Russian Federation

**Keywords:** 5-aminopyrazole, azlactone, elimination, fluorescence, one-pot synthesis, pyrazolo[3,4-*b*]pyridin-6-one

## Abstract

An effective one-pot strategy was developed for the synthesis of 4-arylpyrazolo[3,4-*b*]pyridin-6-ones from pyrazolo[3,4-*b*]pyridin-6-ones, obtained by reacting 5-aminopyrazoles with 4-arylidene-2-phenyloxazol-5(4*H*)-ones (azlactones) under solvent-free conditions, through subsequent elimination of a benzamide molecule in a superbasic medium (*t*-BuOK/DMSO). The fluorescent properties of the synthesized compounds were studied. 4-Arylpyrazolo[3,4-*b*]pyridin-6-ones luminesce in the region of 409–440 nm with a quantum yield of 0.09–0.23 when irradiated with UV light.

## Introduction

**Figure 1:**
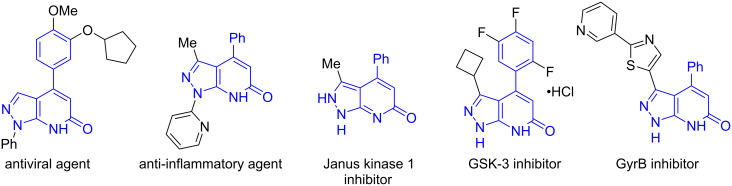
Biologically active 4-arylpyrazolo[3,4-*b*]pyridin-6-ones.

The pyrazolo[3,4-*b*]pyridine scaffold is present in many biologically active compounds [1–12]. Among them, 4-aryl-substituted derivatives should be distinguished, exhibiting antiviral [13] and anti-inflammatory properties [14], being modulators of estrogen-related receptor alpha [15], JAK1 kinase inhibitor [16], GSK3 [17] and GyrB [8] inhibitors (Figure 1).

**Scheme 1:**
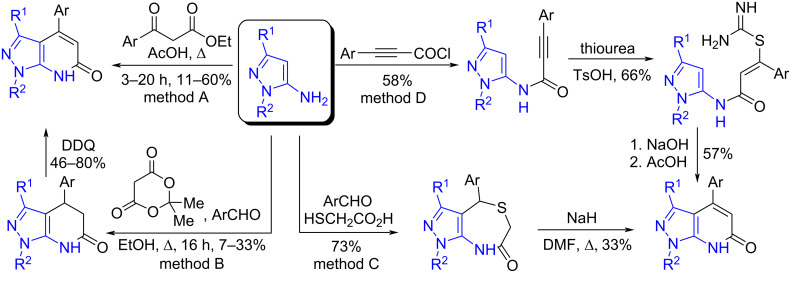
Methods for the synthesis of 4-arylpyrazolo[3,4-*b*]pyridin-6-ones.

Despite the high demand, their synthesis methods are few (Scheme 1). To obtain 4-arylpyrazolo[3,4-*b*]pyridin-6-ones, the only known one-step method is most often used, including the acid-catalyzed condensation of aminopyrazoles with ketoesters [1,16,18] (method A). Its significant disadvantage is the low yields of the target products (11–60%). Yields are also low in two-stage synthesis methods. The first of them is based on the three-component condensation of aminopyrazoles, Meldrum's acid, and aromatic aldehydes, followed by the oxidation of the intermediate with DDQ [13,16,19] (method B). The second one includes the reaction of an aromatic aldehyde with thioglycolic acid and aminopyrazole, followed by the extrusion of sulfur from the resulting thiazepine [20] (method C). The three-stage synthesis of 4-arylpyrazolo[3,4-*b*]pyridin-6-ones, involving the preparation of 3-aryl-*N*-(1*H*-pyrazol-5-yl)propiolamides (method D), also leads to the formation of the target products with low yields [21]. Therefore, the development of a new effective method for the preparation of 4-arylpyrazolo[3,4-*b*]pyridin-6-ones is an urgent task.

## Results and Discussion

One of the rational approaches to the synthesis of fused pyridine derivatives is based on the domino reaction of enamines with azlactones [22–30]. We have previously reported a plausible mechanism of such reactions [22,25]. 1*H*-Pyrazol-5-amines also enter into similar transformations with azlactones in various solvents. The yields of tetrahydro-1*H*-pyrazolo[3,4-*b*]pyridones **3** obtained by this method vary widely [31–33]. Solvent-free reactions are convenient from both economic and environmental points of view. We obtained tetrahydro-1*H*-pyrazolo[3,4-*b*]pyridinone **3a** by heating 5-aminopyrazole **1** with azlactone **2a** in the absence of solvent at 150 °C in 62% yield (Table 1). For compound **3a**, the possibility of benzamide elimination was studied. The benzamide fragment is a poor leaving group; however, in a superbasic medium, we were able to eliminate this group in compound **3a**. In order to select optimal synthesis conditions, we heated compound **3a** in DMSO at temperatures from 90 to 150 °C for 1.5, 3.5 and 6 h in the presence of KOH or *t*-BuOK (Table 1).

**Table 1:** Optimization of reaction conditions^a^.

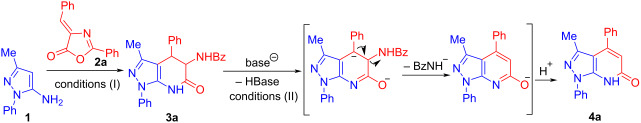			

entry	conditions (I)	conditions (II)	yield of **4a** (%)^b^

1	150 °C, 40 min, (62%)^b^	KOH (1 equiv), DMSO, 90 °C, 6 h	traces
2		KOH (1 equiv), DMSO, 150 °C, 6 h	58^с^
3		KOH (1.5 equiv), DMSO, 150 °C, 3.5 h	63
4		*t-*BuOK (1.5 equiv), DMSO, 150 °C, 1.5 h	81
5^d^	150 °C, 40 min then *t-*BuOK (1.5 equiv), DMSO, 150 °C, 1.5 h		73
6^d^	DMSO, 150 °C, 2.5 h then *t-*BuOK (1.5 equiv), 150 °C, 1.5 h		60

^a^Reaction conditions: **1** (2 mmol), **2a** (2 mmol). ^b^Isolated yield after column chromatography. ^с^Compound **3а** was additionally isolated in 6% yield. ^d^One-pot method.

**Scheme 2:**
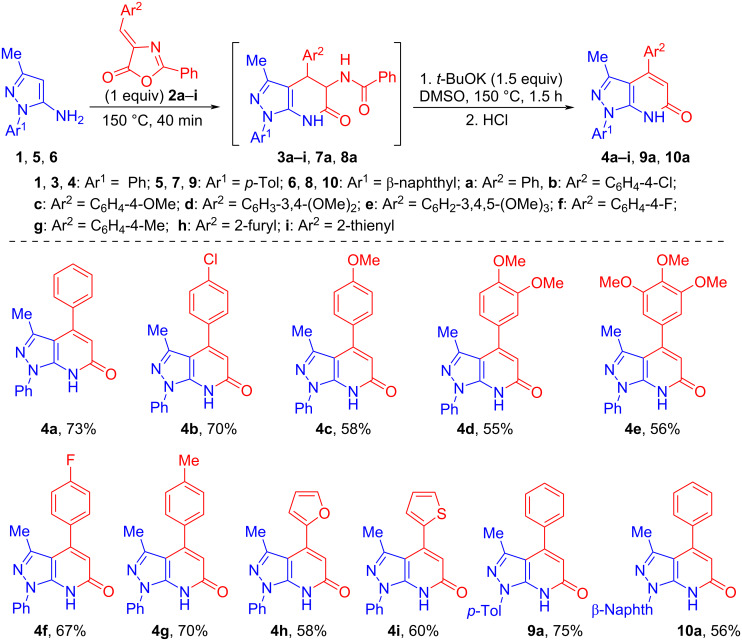
One-pot synthesis of 4-arylpyrazolo[3,4-*b*]pyridin-6-ones **4a**–**i**, **9a**, and **10a**.

The best yield of 4-phenylpyrazolo[3,4-*b*]pyridin-6-one **4а** (81%) was achieved at 150 °C in DMSO containing 1.5 equiv of *t*-BuOK for 1.5 h. Obviously, the preparation of 4-phenylpyrazolo[3,4-*b*]pyridin-6-one **4а** could be carried out as one-pot synthesis, without isolation of the intermediate dihydro derivative **3а**. In this case, the solvent (DMSO) could be added at the stage of obtaining dihydro derivative **3a** or introduced into the reaction together with *t*-BuOK. We have explored both variants. When intermediate **3a** was obtained under solvent-free conditions followed by the addition of *t*-BuOK in DMSO, the yield of pyrazolo[3,4-*b*]pyridin-6-one **4a** was higher (73%, Table 1, entry 5) than when performing the reaction in a solvent (60%, Table 1, entry 6). Therefore, this procedure was used for the synthesis of compounds **4b**–**i**, **9a**, **10a**. The yields of pyrazolo[3,4-*b*]pyridin-6-ones **4a**–**i**, **9a**, **10a** obtained by this method are in the range of 55–75% (Scheme 2).

It should be noted that for compounds containing an electron-donating substituent in the C-4 position, such as 4-methoxyphenyl- (**4c**), 3,4-dimethoxyphenyl- (**4d**), 3,4,5-trimethoxyphenyl- (**4e**), 2-furyl- (**4h**) and 2-thienyl- (**4i**), the product yields are reduced to 55–60% (Scheme 2).

All the compounds obtained are colorless crystalline substances. When dissolved, they produce colorless solutions exhibiting distinct fluorescent properties with blue emission when exposed to UV light. We recorded absorption and fluorescence spectra of ethanolic solutions of compounds **4a**–**i**, **9a**, and **10a**. The emission and absorption spectra of all the compounds differ slightly from each other. Their spectral parameters are presented in Table 2.

**Table 2:** Data of absorption and fluorescence spectra of compounds **4a**–**i**, **9a**, and **10a**.^a^

Compound	UV–vis				Photoluminescence	
	^max^λ_abs,_ nm	ε, 10^3^, M^–1^·cm^–1^ (λ, nm)	λ_ex_, nm	^max^λ_em_, nm	Stokes shift, nm; eV	Quantum yield Φ_fl_^b^

**4a**	260; 302	30.3 ± 0.7 (260)	300; 320	419	117; 1.15	0.22 ± 0.01
**4b**	260; 302	38.3 ± 0.7 (260)	300; 320	428	126; 1.21	0.23 ± 0.01
**4c**	262; 302	22.2 ± 0.8 (262)	300; 320	409	107; 1.07	0.16 ± 0.01
**4d**	260; 301	35.1 ± 0.9 (260)	300; 320	414	113; 1.12	0.15 ± 0.01
**4e**	262; 301	22.7 ± 0.9 (262)	300; 320	416	115; 1.14	0.18 ± 0.01
**4f**	260; 302	27.6 ± 0.8 (260)	300; 320	415	113; 1.12	0.20 ± 0.01
**4g**	261; 300	41.5 ± 0.9 (261)	300; 320	411	111; 1.12	0.20 ± 0.01
**4h**	265; 305	32.4 ± 1.0 (265)	300; 310	421	116; 1.12	0.23 ± 0.01
**4i**	263; 301	26.2 ± 0.8 (263)	300; 310	431	130; 1.24	0.09 ± 0.00
**9a**	259; 303	40.0 ± 0.9 (261)	305	433	130; 1.23	0.19 ± 0.01
**10a**	261; 288	34.9 ± 0.5 (259)	290	440	152; 1.49	0.11 ± 0.01

^a^In EtOH solution, *c* = 1.0·10^−5^ mol·L^−1^. ^b^Quantum yield determined relative to quinine sulfate standard in 0.5 M H_2_SO_4_ (Ф_f_ = 0.546).

**Figure 2:**
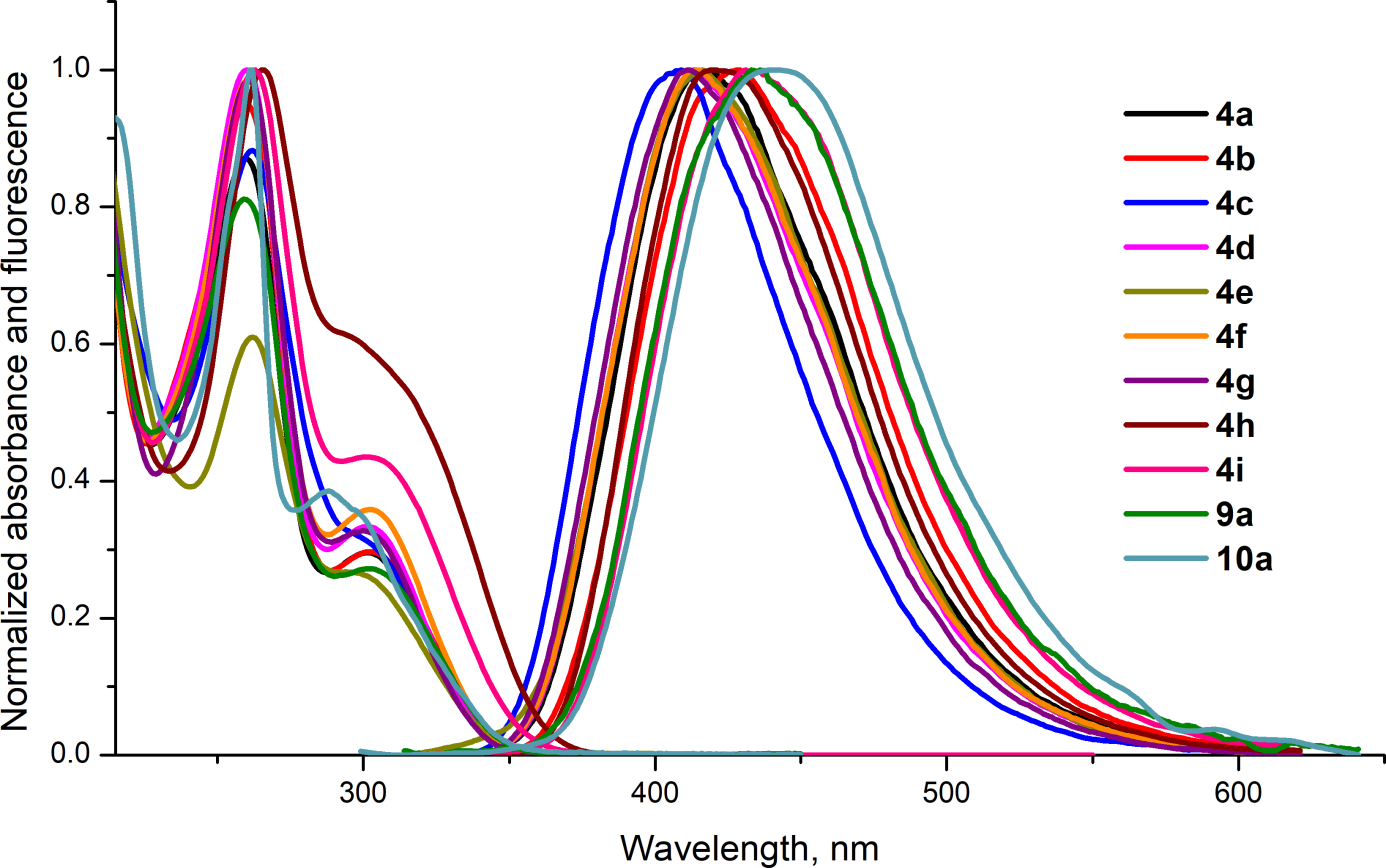
Normalized absorption and fluorescence spectra of solutions of compounds **4a**–**i**, **9a**, and **10a** in EtOH.

In the UV spectra of ethanolic solutions of compounds **4a–i**, **9a**, and **10a**, a band with a maximum at 260–265 nm is observed, which has a shoulder at 300–305 nm. These signals seem to correspond to π–π* and n–π* transitions. In the luminescence spectra of compounds **4a**–**i**, **9a**, and **10a**, there is one broadened band with an emission maximum at 409–440 nm (Figure 2). Their diluted alcohol solutions luminesce with a quantum yield of 0.09–0.23. Pyrazolo[3,4-*b*]pyridinones **4a**–**i**, **9a**, and **10a** are characterized by an abnormally high Stokes shift (107–152 nm, 1.07–1.49 eV, Table 2). Such luminophores, which are colorless in daylight but become colored when irradiated with UV light, are used in forensics, in protection against forgery of banknotes, securities, and other important documents [34].

## Conclusion

In summary, we developed a simple one-pot synthesis of 4-arylpyrazolo[3,4-*b*]pyridin-6-ones, based on the solvent-free reaction of the available starting compounds 5-aminopyrazoles **1**, **5**, **6** and azlactones **2a**–**i**, followed by heating the resulting intermediate in DMSO in the presence of *t*-BuOK. Photophysical properties of the obtained compounds were studied.

## Supporting Information

File 1 Experimental procedures, characterization data, and ^1^H and ^13^C NMR spectra for all new compounds.
