# Supplementary material for: New one-pot synthesis of 4-arylpyrazolo[3,4-b]pyridin-6-ones based on 5-aminopyrazoles and azlactones
Source: Beilstein J Org Chem. 2023 Aug 2;19:1155–60. doi: 10.3762/bjoc.19.83 (PMC10407779; doi:10.3762/bjoc.19.83)
Supplement: File 1 — Experimental procedures, characterization data, and 1H and 13C NMR spectra for all new compounds. [file Beilstein_J_Org_Chem-19-1155-s001.pdf]

BEILSTEIN JOURNAL OF ORGANIC CHEMISTRY

Supporting Information

for

New one-pot synthesis of 4-arylpyrazolo[3,4-*b*]pyridin-6-ones based on 5-aminopyrazoles and azlactones

Vladislav Yu. Shuvalov, Ekaterina Yu. Vlasova, Tatyana Yu. Zheleznova and Alexander S. Fisyuk

Beilstein J. Org. Chem. **2023,** *19,* 1155–1160. doi:10.3762/bjoc.19.83

Experimental procedures, characterization data, and ^{1}H and ^{13}C NMR spectra for all new compounds

License and Terms: This is a supporting information file under the terms of the Creative Commons Attribution License (https://creativecommons.org/licenses/by/4.0). Please note that the reuse, redistribution and reproduction in particular requires that the author(s) and source are credited and that individual graphics may be subject to special legal provisions.
The license is subject to the *Beilstein Journal of Organic Chemistry* terms and conditions: (https://www.beilstein-journals.org/bjoc/terms)

Table of contents

General information..S2

Compounds characterization and analytical data..S3

References...S8

Copies of NMR spectra...S10

General information

^{1}H and ^{13}C NMR spectra were acquired on a Bruker DRX-400 spectrometer (working frequencies 400 and 100 MHz, respectively) in $CDCl_3$ or DMSO-d_6, with the residual solvent signals ($CDCl_3$: 7.26 ppm for ^{1}H nuclei and 77.0 ppm for ^{13}C nuclei; DMSO-d_6: 2.50 ppm for ^{1}H nuclei and 39.5 ppm for ^{13}C nuclei) serving as internal standards. All chemical shifts (δ) are reported in ppm and coupling constants (*J*) in Hz. Absorption spectra were recorded on a PerkinElmer Lambda 750 diode array spectrophotometer, photoluminescence spectra were recorded on a Cary Eclipse fluorescence spectrophotometer. In both cases, the test compounds were dissolved in EtOH so that the concentration of the resulting solutions was lower than 10^{-5} mol/dm^3. The molar light absorption coefficient was determined according to the described method [1,2]. The quantum yield was determined relative to quinine sulfate in 0.5 M H_2SO_4 (Φ_f 0.546) using the comparison method [3,4]. Infra-red spectra were recorded on an Infralum FT-801 spectrometer for others in KBr pellets. The elemental analyses were carried out on a Carlo Erba 1106 CHN analyzer. The melting points were determined on a Reach devices RD-MP. Monitoring of the reaction progress and assessment of the purity of synthesized compounds were done by TLC on Silufol UV-254 plates, eluents $CHCl_3$–EtOAc, 10:1. Visualization of plates was done under UV light. Silica gel 60 (0.063–0.200 mm, Macherey–Nagel) was used for column chromatography.

The starting compounds **1** [5], **2a**–**i** [6-10], **5** [11] and **6** [12] were prepared following the reported methods.

Compounds characterization and analytical data

_N_-(3-Methyl-6-oxo-1,4-diphenyl-4,5,6,7-tetrahydro-1_H_-pyrazolo[3,4-_b_]pyridin-5-

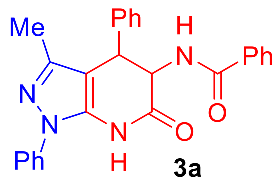

3a

yl)benzamide (3a). A mixture of 3-methyl-1-phenyl-1*H*-pyrazol-5-amine (**1**, 346 mg, 2 mmol) and 4-benzylidene-2-phenyloxazol-5(4*H*)-one (**2a**, 498 mg, 2 mmol) was heated at 150 °C for 40 min, after cooling to room temperature, the product was purified by column chromatography on silica gel (eluent, $CHCl_3$–EtOAc, 10:1). Yield: 523 mg (62%), colorless crystals, m.p. >250 °C (*i*-PrOH) (m.p. 268–270 °C (EtOH) [13]). IR (KBr): 3295, 3194, 3062, 3032, 2911, 1698, 1693, 1642, 1601, 1582, 1547, 1496, 1457, 1374, 1357, 1335, 1267, 1255, 1184, 1113, 1075, 1054, 1026, 1002, 916, 819, 819, 800, 754, 694, 660 cm^{-1}. ^1H NMR (400 MHz, DMSO-d_6) δ*:* 1.46 (s, 3H), 4.40 (d, *J* = 11.9 Hz, 1H), 5.05 (dd, *J* = 11.9, 9.0 Hz, 1H), 7.20-7.26 (m, 1H), 7.31-7.39 (m, 5H), 7.40-7.45 (m, 2H), 7.48-7.53 (m, 3H), 7.55-7.59 (m, 2H), 7.71-7.74 (m, 2H), 8.68 (d, *J* = 9.0 Hz, 1H), 10.79 (s, 1H), ^{13}C NMR (100 MHz, DMSO-d_6) δ*:* 12.5, 41.4, 56.0, 102.3, 122.9, 126.8, 127.1, 127.2, 128.2, 128.3, 128.5, 129.2, 131.3, 134.1, 137.3, 137.9, 140.1, 145.5, 166.4, 168.5. Anal. Calcd for $C_{26}H_{22}N_4O_2$: C 73.92, H 5.25, N 13.26. Found: 73.80, H 5.30, N 13.15.

General procedure for the synthesis of 3-methyl-1-phenyl-1,7-dihydro-6*H*-pyrazolo[3,4-*b*]pyridin-6-one (4a-i). A mixture of 3-methyl-1-phenyl-1*H*-pyrazol-5-amine (**1**, 346 mg, 2 mmol) and azlactones **2a–i** (2 mmol) was heated at 150 °C for 40 min. Then the mixture was cooled and DMSO (4 mL) and *t*-BuOK (336 mg, 3 mmol) was added, after which the resulting mixture was heated at 150 °C for 1.5 h. The reaction mixture was cooled, poured into H_2O (30 mL), and 10% HCl was added on cooling to pH ≈ 3. The precipitate formed was filtered off, dried, and purified by column chromatography on silica gel (eluent, $CHCl_3$–EtOAc, 10:1).

3-Methyl-1,4-diphenyl-1,7-dihydro-6*H*-pyrazolo[3,4-*b*]pyridin-6-one (4a). Yield: 439 mg

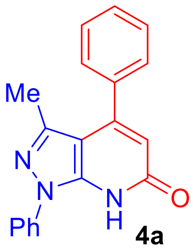

4a

(73%), colorless crystals, m.p. 194-195 °C (m.p. 193-194 °C [14]). IR (KBr): 3056, 1634, 1599, 1586, 1568, 1512, 1503, 1495, 1472, 1457, 1443, 1426, 1402, 1383, 1352, 1316, 1258, 1248, 1213, 1161, 1121, 1082, 1021, 986, 889, 860, 847, 776, 762, 725, 706, 696, 679, 637 cm^{-1}. ^{1}H NMR (400 MHz, $CDCl_3$) δ*:* 2.09 (s, 3H), 6.34 (s, 1H), 7.14-7.18 (m, 1H), 7.26-7.32 (m, 2H), 7.43-7.52 (m, 5H), 7.61-7.67 (m, 2H), 11.01 (br.s, 1H). ^{13}C NMR (100 MHz, $CDCl_3$) δ*:* 14.7, 107.3, 110.2, 122.2, 126.8, 128.3, 128.4, 129.0, 129.0, 137.1, 137.5, 143.9, 144.0, 150.6, 163.6. Anal. Calcd for $C_{19}H_{15}N_3O$: C 75.73, H 5.02, N 13.94. Found: C 75.88, H 5.14, N 13.80.

4-(4-Chlorophenyl)-3-methyl-1-phenyl-1,7-dihydro-6*H*-pyrazolo[3,4-*b*]pyridin-6-one (4b).

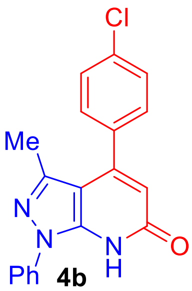

4b

Yield: 470 mg (70%), colorless crystals, m.p. 228-229 °C. IR (KBr): 3056, 1647, 1642, 1595, 1576, 1503, 1429, 1383, 1348, 1281, 1262, 1159, 1121, 1090, 1015, 988, 887, 862, 830, 760, 731, 714, 691, 675 cm^{-1}. ^{1}H NMR (400 MHz, $CDCl_3$) δ*:* 2.10 (s, 3H), 6.30 (s, 1H), 7.16-7.21 (m, 1H), 7.29-7.34 (m, 2H), 7.36-7.41 (m, 2H), 7.46-7.52 (m, 2H), 7.60-7.65 (m, 2H), 10.84 (br.s, 1H). ^{13}C NMR (100 MHz, $CDCl_3$) δ*:* 14.8, 107.0, 110.3, 122.2, 127.0, 128.7, 129.1, 129.7, 135.2, 135.5, 137.4, 143.8, 143.9, 149.2, 163.4. Anal. Calcd for $C_{19}H_{14}ClN_3O$: C 67.96, H, 4.20, N 12.51. Found: C 67.82, H, 4.11, N 12.34.

4-(4-Methoxyphenyl)-3-methyl-1-phenyl-1,7-dihydro-6*H*-pyrazolo[3,4-*b*]pyridin-6-one

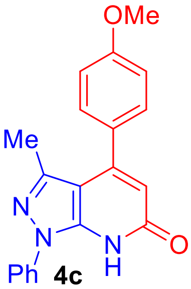

4c

(4c). Yield: 384 mg (58%), colorless crystals, m.p. 188-189 °C. IR (KBr): 3063, 1632, 1597, 1586, 1574, 1503, 1387, 1356, 1252, 1211, 1159, 1090, 1015, 980, 889, 858, 835, 764, 733, 696 cm^{-1}. ^{1}H NMR (400 MHz, $CDCl_3$) δ*:* . 2.15 (s, 3H), 3.88 (s, 3H), 6.30 (s, 1H), 6.99-7.04 (m, 2H), 7.11-7.17 (m, 1H), 7.26-7.31 (m, 2H), 7.37-7.41 (m, 2H), 7.59-7.64 (m, 2H), 10.85 (br.s, 1H). ^{13}C NMR (100 MHz, $CDCl_3$) δ*:* 15.0, 55.3, 107.3, 110.1, 113.8, 122.2, 126.9, 129.0, 129.4, 129.7, 137.5,

143.8, 144.1, 150.5, 160.3, 163.6. Anal. Calcd for $C_{20}H_{17}N_3O_2$: C 72.49, H 5.17, N 12.68. Found: C 72.34, H 5.13, N 12.79.

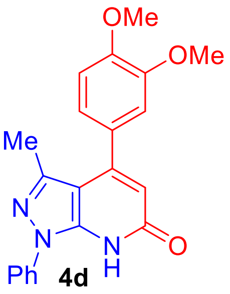

4d

4-(3,4-Dimethoxyphenyl)-3-methyl-1-phenyl-1,7-dihydro-6*H*-pyrazolo[3,4-*b*]pyridin-6-one (4d). Yield: 397 mg (55%), colorless crystals, m.p. 243-244 °C. IR (KBr): 3069, 1634, 1607, 1599, 1582, 1514, 1429, 1356, 1221, 1159, 1115, 1015, 980, 862, 839, 799, 733, 698 cm^{-1}. ^{1}H NMR (400 MHz, DMSO-d_6) δ*:* 2.19 (s, 3H), 3.82 (s, 3H), 3.83 (s, 3H), 6.47 (s, 1H), 7.04-7.11 (m, 2H), 7.13 (s, 1H), 7.28 (t, *J* = 7.4 Hz, 1H), 7.51 (t, *J* = 7.8 Hz, 2H), 8.21 (d, *J* = 7.8 Hz, 2H),11.39 (br.s, 1H). ^{13}C NMR (100 MHz, DMSO-d_6) δ*:* 15.3, 55.5, 55.6, 106.0, 109.2, 111.4, 112.5, 120.5, 121.3, 125.2, 128.9, 129.4, 139.3, 142.3, 148.3, 148.4, 149.3, 150.0, 163.4. Anal. Calcd for $C_{21}H_{19}N_3O_3$: C 69.79, H 5.30, N 11.63. Found: C 69.60, H 5.19, N 11.48.

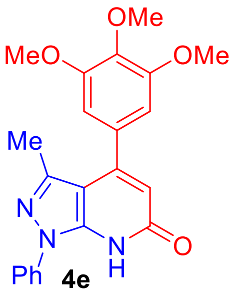

4e

3-Methyl-1-phenyl-4-(3,4,5-trimethoxyphenyl)-1,7-dihydro-6*H*-pyrazolo[3,4-*b*]pyridin-6-one (4e). Yield: 438 mg (56%), colorless crystals, m.p. 184-185 °C. IR (KBr): 3061, 3001, 2928, 2835, 1644, 1582, 1574, 1509, 1468, 1455, 1410, 1356, 1325, 1237, 1179, 1131, 1003, 828, 760, 731, 693 cm^{-1}. ^{1}H NMR (400 MHz, $CDCl_3$) δ*:* 2.19 (s, 3H), 3.89 (s, 6H), 3.92 (s, 3H), 6.34 (s, 1H), 6.66 (s, 2H), 7.18-7.23 (m, 1H), 7.31-7.36 (m, 2H), 7.65-7.68 (m, 2 H), 10.70 (br.s, 1H). ^{13}C NMR (100 MHz, $CDCl_3$) δ*:* 15.0, 56.2, 61.0, 105.8, 107.2, 110.1, 122.2, 127.0, 129.2, 132.5, 137.5, 138.7, 143.9, 144.0, 150.5, 153.1, 163.4. Anal. Calcd for $C_{22}H_{21}N_3O_4$: C 67.51, H 5.41, N 10.74. Found: C 67.66, H 5.19, N 10.83.

4-(4-Fluorophenyl)-3-methyl-1-phenyl-1,7-dihydro-6*H*-pyrazolo[3,4-*b*]pyridin-6-one (4f).

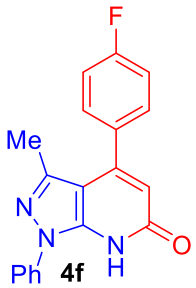

Yield: 427 mg (67%), colorless crystals, m.p. 239-240 °C. IR (KBr): 3075, 1632, 1582, 1514, 1429, 1389, 1356, 1266, 1252, 1221, 1157, 1115, 1015, 780, 924, 899, 862, 857, 839, 799, 764, 731, 696, 675, 569 cm^{-1}. ^{1}H NMR (400 MHz, $CDCl_3$) δ*:* 2.10 (s, 3H), 6.31 (s, 1H), 7.16-7.24 (m, 3H), 7.31-7.37 (m, 2H), 7.39-7.45 (m, 2H), 7.63-7.67 (m, 2H), 10.50 (br.s, 1H). ^{13}C NMR (100 MHz, $CDCl_3$) δ*:* 14.8, 107.2, 110.3, 115.5 (d*, J* = 21.6 Hz), 122.3, 127.0, 129.2, 130.2 (d*, J* = 7.8 Hz), 133.1 (d*, J* = 3.5 Hz), 137.5, 143.8, 144.0, 149.5, 163.2 (d*, J* = 248.8 Hz), 163.3. Anal. Calcd for $C_{19}H_{14}FN_3O$: C 71.46, H 4.42, N 13.16. Found: C 71.32, H 4.54, N 12.99.

3-Methyl-1-phenyl-4-(*p*-tolyl)-1,7-dihydro-6*H*-pyrazolo[3,4-*b*]pyridin-6-one (4g). Yield:

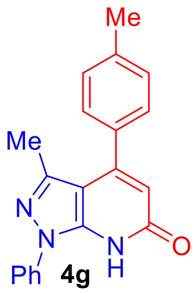

441 mg (70%), colorless crystals, m.p. 217-218 °C. IR (KBr): 3060, 1642, 1584, 1516, 1497, 1428, 1383, 1348, 1266, 1210, 1183, 1159, 1119, 1011, 984, 851, 824, 762, 729, 693 cm^{-1}. ^{1}H NMR (400 MHz, $CDCl_3$) δ*:* 2.12 (s, 3H), 2.45 (s, 3H), 6.31 (s, 1H), 7.12-7.17 (m, 1H), 7.26-7.36 (m, 6H), 7.59-7.63 (m, 2H), 10.90 (br.s, 1H). ^{13}C NMR (100 MHz, $CDCl_3$) δ*:* 14.9, 21.3, 107.2, 110.2, 122.2, 126.9, 128.3, 129.0 (3C), 134.2, 137.4, 139.0, 143.8, 144.1, 150.8, 163.6. Anal. Calcd for $C_{20}H_{17}N_3O$: C 76.17, H 5.43, N 13.32. Found: C 76.54, H 5.53, N 13.12.

4-(Furan-2-yl)-3-methyl-1-phenyl-1,7-dihydro-6*H*-pyrazolo[3,4-*b*]pyridin-6-one (4h).

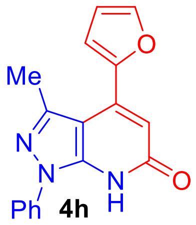

Yield: 338 mg (58%), colorless crystals, m.p. 188-189 °C. IR (KBr): 3048, 1597, 1580, 1557, 1511, 1493, 1418, 1387, 1343, 1277, 1267, 1204, 1175, 1157, 1140, 1038, 1015, 957, 858, 820, 776, 764, 752, 727, 696, 658, 658, 598 cm^{-1}. ^{1}H NMR (400 MHz, $CDCl_3$) δ*:* 2.57 (s, 3H), 6.57 (m, 2H), 6.92 (d, *J* = 3.3 Hz, 1H), 7.12 (t, *J* = 7.4 Hz, 1H), 7.24 (t, *J* = 7.7 Hz, 2H), 7.57-7.61 (m, 2H), 7.66 (d, *J* = 1.2 Hz, 1H), 10.54 (br.s, 1H). ^{13}C NMR (100 MHz, $CDCl_3$) δ*:* 16.0, 105.2, 106.4, 111.8, 112.2, 122.2, 126.9, 128.9, 137.3, 137.7, 144.0, 144.1, 145.1, 150.4, 163.4. Anal. Calcd for $C_{17}H_{13}N_3O_2$: C 70.09, H 4.50, N 14.42. Found: C 70.19, H 4.41, N 14.28.

3-Methyl-1-phenyl-4-(thiophen-2-yl)-1,7-dihydro-6*H*-pyrazolo[3,4-*b*]pyridin-6-one (4i).

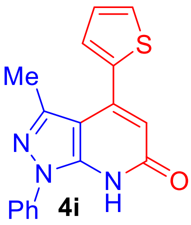
4i

Yield: 368 mg (60%), colorless crystals, m.p. 190-191 °C. IR (KBr): 3069, 1642, 1597, 1572, 1511, 1497, 1439, 1385, 1354, 1333, 1219, 1213, 1183, 1138, 1080, 1038, 1026, 1013, 976, 909, 849, 806, 799, 758, 727, 712, 694, 666 cm^{-1}. ^{1}H NMR (400 MHz, DMSO-d_6) δ*:* 2.32 (s, 3H), 6.54 (s, 1H), 7.23-7.31 (m, 2H), 7.45-7.54 (m, 3H), 7.78 (d, *J* = 5.0 Hz, 1H), 8.17 (d, *J* = 7.7 Hz, 2H), 11.36 (br.s, 1H). ^{13}C NMR (100 MHz, DMSO-d_6) δ*:* 15.3, 106.6, 108.6, 120.7, 125.5, 127.9, 128.2, 128.9, 129.2, 137.2, 139.1, 140.6, 142.1, 150.1, 163.4. Anal. Calcd for $C_{17}H_{13}N_3OS$: C 66.43, H 4.26, N 13.67. Found: C 66.33, H 4.38, N 13.53.

3-Methyl-4-phenyl-1-(*p*-tolyl)-1,7-dihydro-6*H*-pyrazolo[3,4-*b*]pyridin-6-one (9a). Yield: 473 mg (75%), colorless crystals, m.p. 188-189 °C. IR (KBr): 3063, 1640, 1605, 1593, 1582,

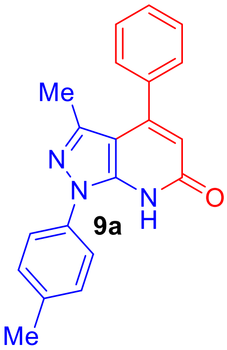
9a

1566, 1520, 1493, 1445, 1381, 1347, 1302, 1286, 1271, 1246, 1217, 1179, 1159, 1144, 1109, 1078, 1021, 990, 924, 860, 845, 820, 795, 766, 702, 667, 642, 615 cm^{-1}. ^{1}H NMR (400 MHz, $CDCl_3$) δ*:* 2.08 (s, 3H), 2.25 (s, 3H), 6.34 (s, 1H), 7.05 (d, *J* = 8.2 Hz, 2H), 7.42-7.55 (m, 7H), 10.91 (br.s, 1H). ^{13}C NMR (100 MHz, $CDCl_3$) δ*:* 14.7, 21.0, 106.9, 110.3, 122.1, 128.3, 128.4, 129.0, 129.5, 134.8, 136.9, 137.2, 143.5, 143.7, 150.7, 163.6. Anal. Calcd for $C_{20}H_{17}N_3O$: C 76.17, H 5.43, N 13.32. Found: C 76.48, H 5.50, N 13.16.

3-Methyl-1-(naphthalen-2-yl)-4-phenyl-1,7-dihydro-6*H*-pyrazolo[3,4-*b*]pyridin-6-one

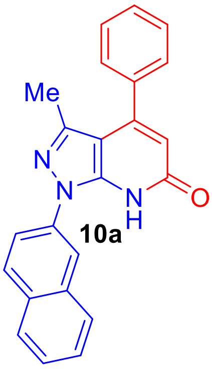
10a

(10a). Yield: 394 mg (56%), colorless crystals, m.p. 224-225 °C. IR (KBr): 3050, 1640, 1634, 1603, 1566, 1514, 1499, 1474, 1443, 1387, 1352, 1286, 1557, 1131, 1075, 1017, 887, 858, 814, 774, 747, 749, 760 cm^{-1}. ^{1}H NMR (400 MHz, $CDCl_3$) δ*:* 1.90 (s, 3H), 6.43 (s, 1H), 7.31-7.43 (m, 4H), 7.49-7.54 (m, 4H), 7.58 (dd, *J* = 8.8, 2.2 Hz, 1H), 7.65-7.70 (m, 2H), 7.93 (d, *J* = 1.8 Hz, 1H), 12.04 (br.s, 1H). ^{13}C NMR (100 MHz, $CDCl_3$) δ*:* 14.7, 107.5, 109.7, 118.3, 120.8, 125.7, 126.5, 127.4, 127.5, 128.3, 128.4, 128.8, 129.0, 131.5, 132.7, 134.8,

137.0, 143.7, 143.9, 150.8, 164.3. Anal. Calcd for $C_{23}H_{17}N_3O$: C 78.61, H 4.88, N 11.96. Found: C 78.47, H 4.92, N 12.01.

References

1. Kumar, A.; Singh, S.; Singh Mudahar, G.; Singh Thind, K. *Radiat. Phys. Chem.* **2006**, *75*, 737–740. doi:10.1016/j.radphyschem.2005.12.035
2. Williams, A. T. R.; Winfield, S. A.; Miller, J. N. *Analyst* **1983**, *108*, 1067. doi:10.1039/an9830801067
3. Allen, M. W. *Measurement of Fluorescence Quantum Yields*; Thermo Fisher Scientific: Madison. Technical note: 52019.
4. Brouwer, A. M. *Pure Appl. Chem.* **2011**, *83*, 2213–2228. doi:10.1351/PAC-REP-10-09-31
5. Ganesan, A.; Heathcock, C. H. *J. Org. Chem.* **1993**, *58*, 6155–6157. doi:10.1021/jo00074a058
6. Shuvalov, V. Yu.; Rozhkova, Yu. S.; Plekhanova, I. V; Kostyuchenko, A. S.; Shklyaev, Yu. V; Fisyuk, A. S. *Chem. Heterocycl. Compd.* **2022**, *58*, 7–14. doi:10.1007/s10593-022-03050-5
7. Shuvalov, V. Yu.; Chernenko, S. A.; Shatsauskas, A. L.; Samsonenko, A. L.; Dmitriev, M. V; Fisyuk, A. S. *Chem. Heterocycl. Compd.* **2021**, *57*, 764–771. doi:10.1007/s10593-021-02980-w
8. Pinheiro, D. L. J.; Nielsen, M. *J. Org. Chem.* **2022**, *87*, 5419–5423. doi:10.1021/acs.joc.1c02975
9. Ghanim, A. M.; Rezq, S.; Ibrahim, T. S.; Romero, D. G.; Kothayer, H. *Eur. J. Med. Chem.* **2021**, *219*, 113457. doi:10.1016/j.ejmech.2021.113457

10. Marra, I. F. S.; de Almeida, A. M.; Silva, L. P.; de Castro, P. P.; Corrêa, C. C.; Amarante, G. W. *J. Org. Chem.* **2018**, *83*, 15144–15154. doi:10.1021/acs.joc.8b02430
11. Nam, N. L.; Grandberg, I. I.; Sorokin, V. I. *Chem. Heterocycl. Compd*. **2000,** *36,* 281–283. doi:10.1007/BF02256864
12. Li, Y.; Huang, X.; He, J.; Peng, S.; Wang, J.; Lang, M. *Adv. Synth. Catal*. **2023**, *365,* 490–495. doi:10.1002/adsc.202201335
13. Shi, F.; Zhang, J.; Tu, S.; Jia, R.; Zhang, Y.; Jiang, B.; Jiang, H. *J. Heterocycl. Chem*. **2007,** *44,* 1013–1017. doi:10.1002/jhet.5570440506
14. Minami, S.; Tomita, M.; Kawaguchi, K. *Chem. Pharm. Bull.* **1972,** *20,* 1716–1728. doi:10.1248/cpb.20.1716

Copies of NMR spectra

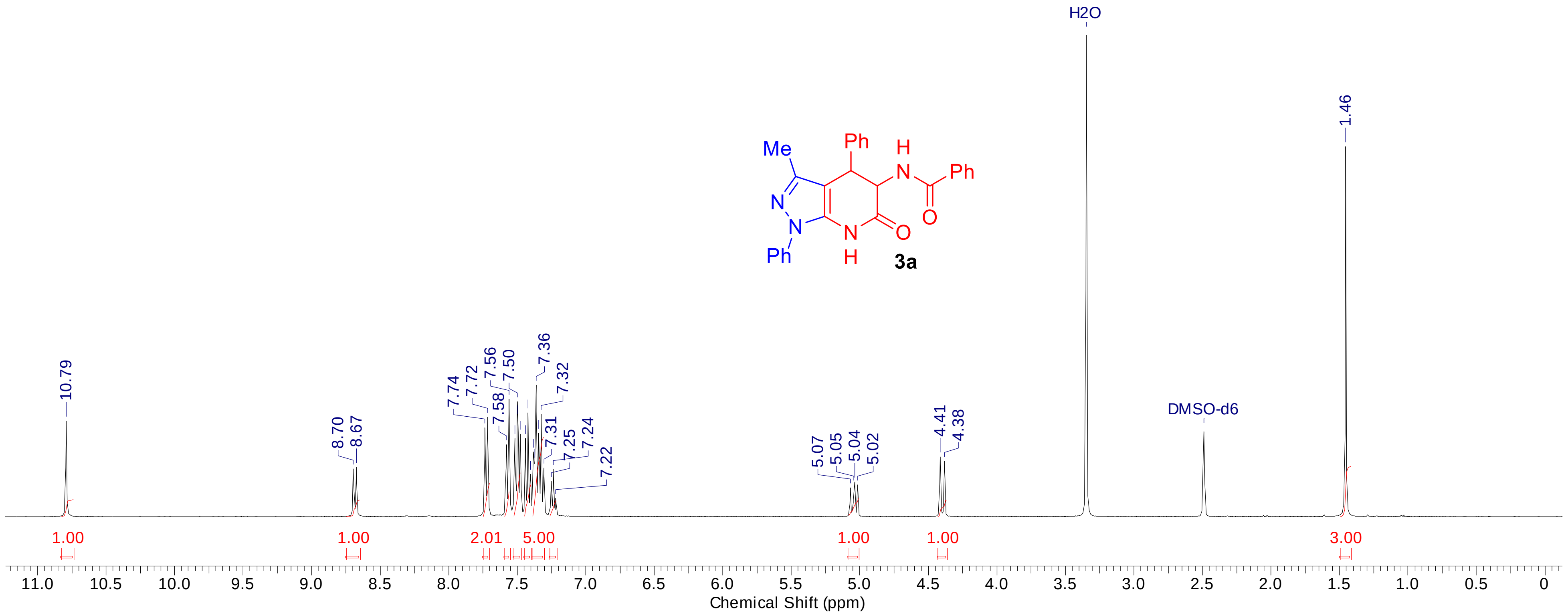

Figure S1: ^{1}H NMR spectrum of compound **3a** (DMSO-d_6).

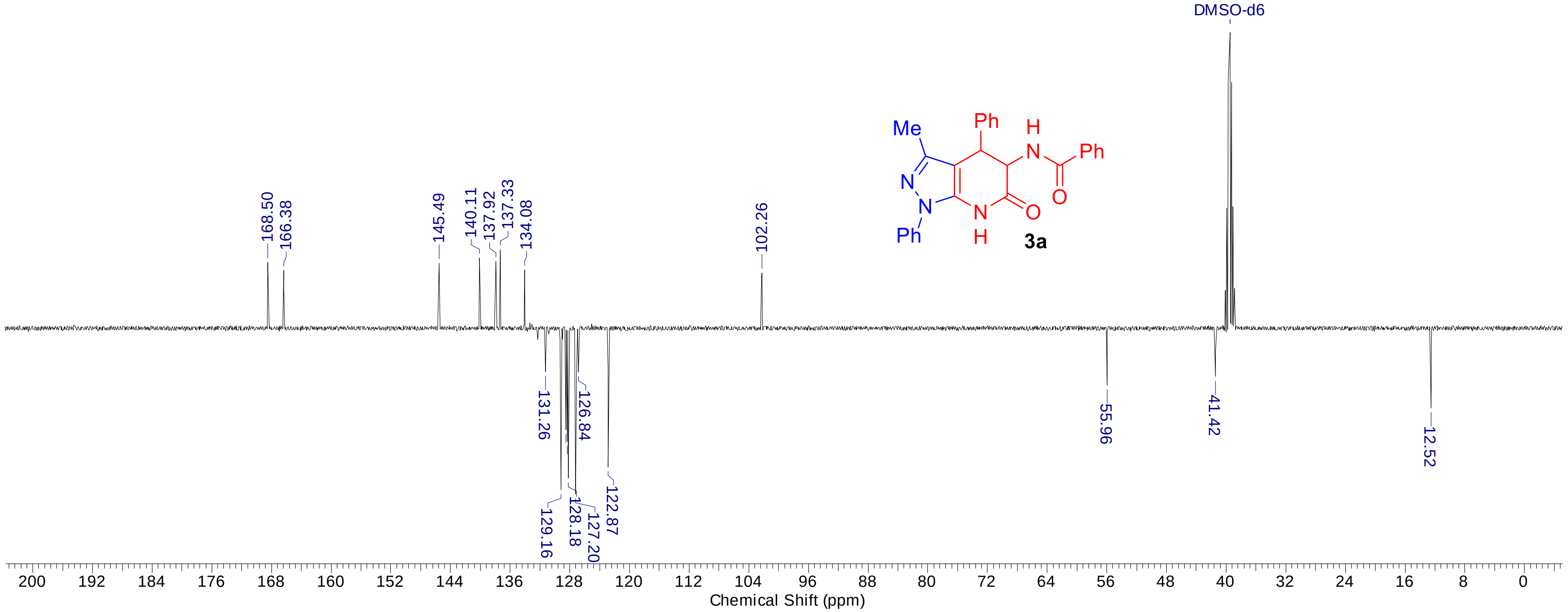

Figure S2: ^{13}C NMR spectrum of compound **3a** (DMSO-d_6).

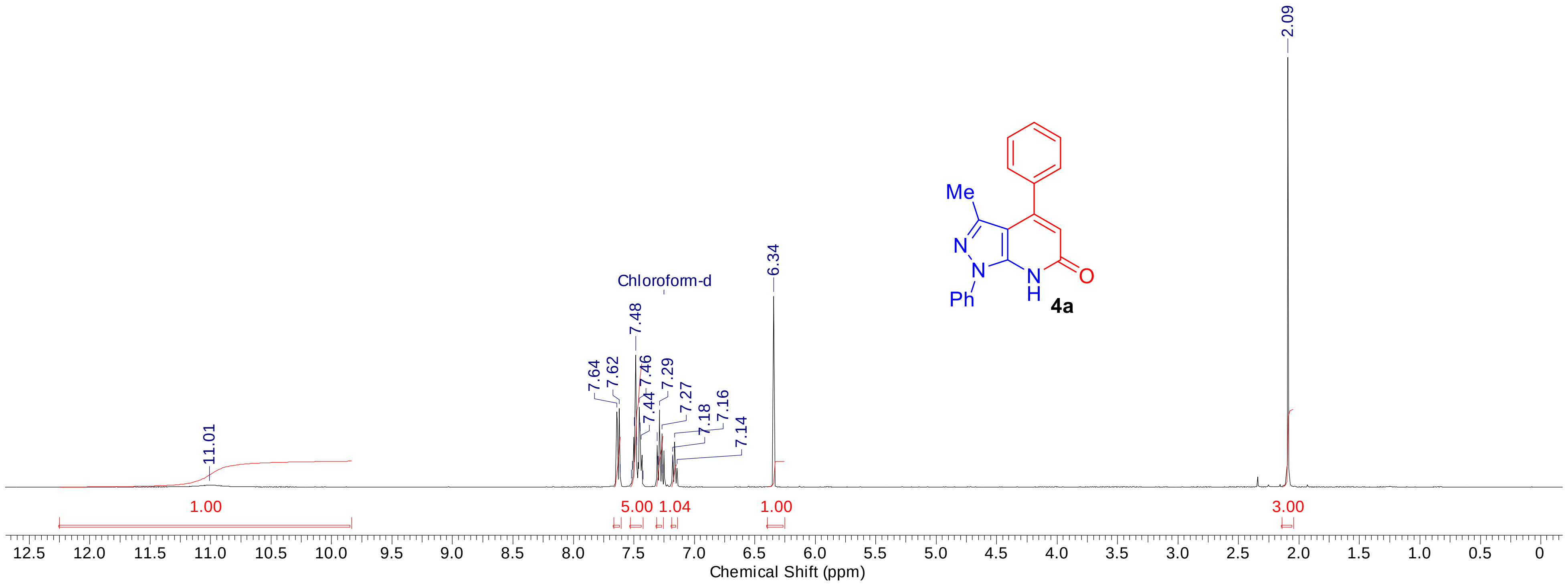

Figure S3: ^{1}H NMR spectrum of compound **4a** ($CDCl_3$).

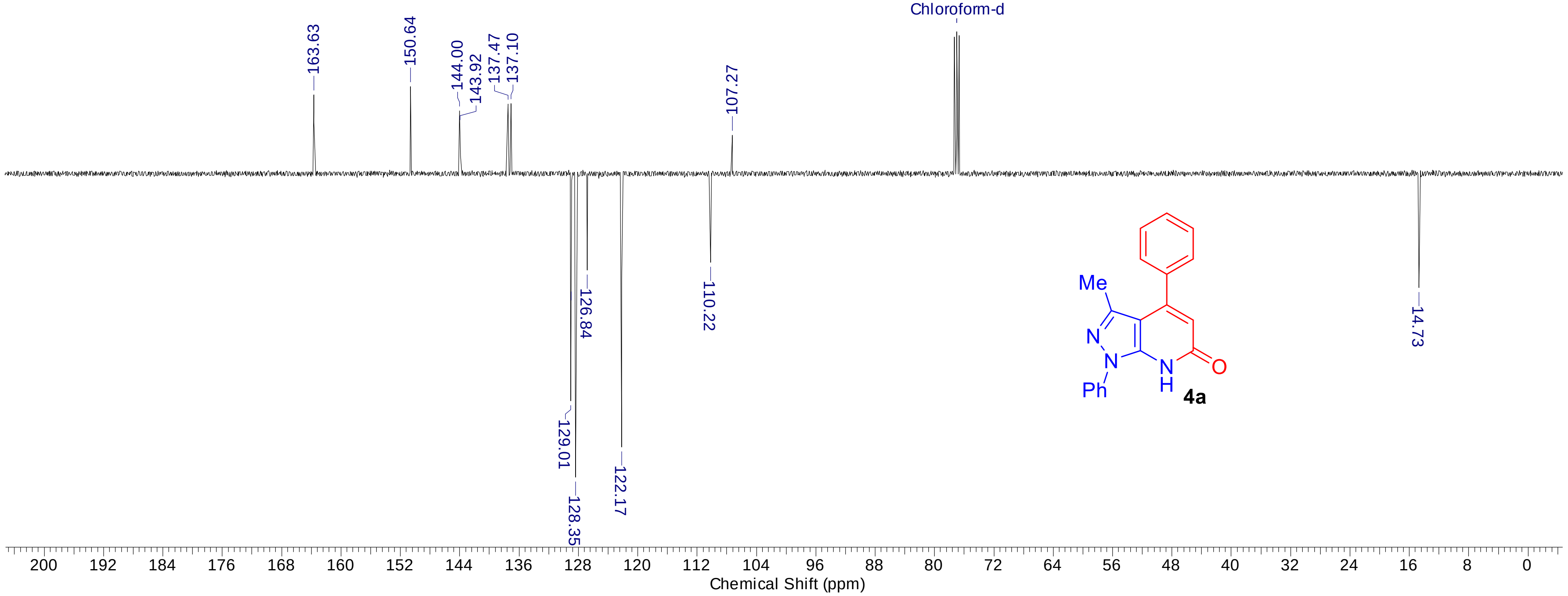

Figure S4: ^{13}C NMR spectrum of compound **4a** ($CDCl_3$).

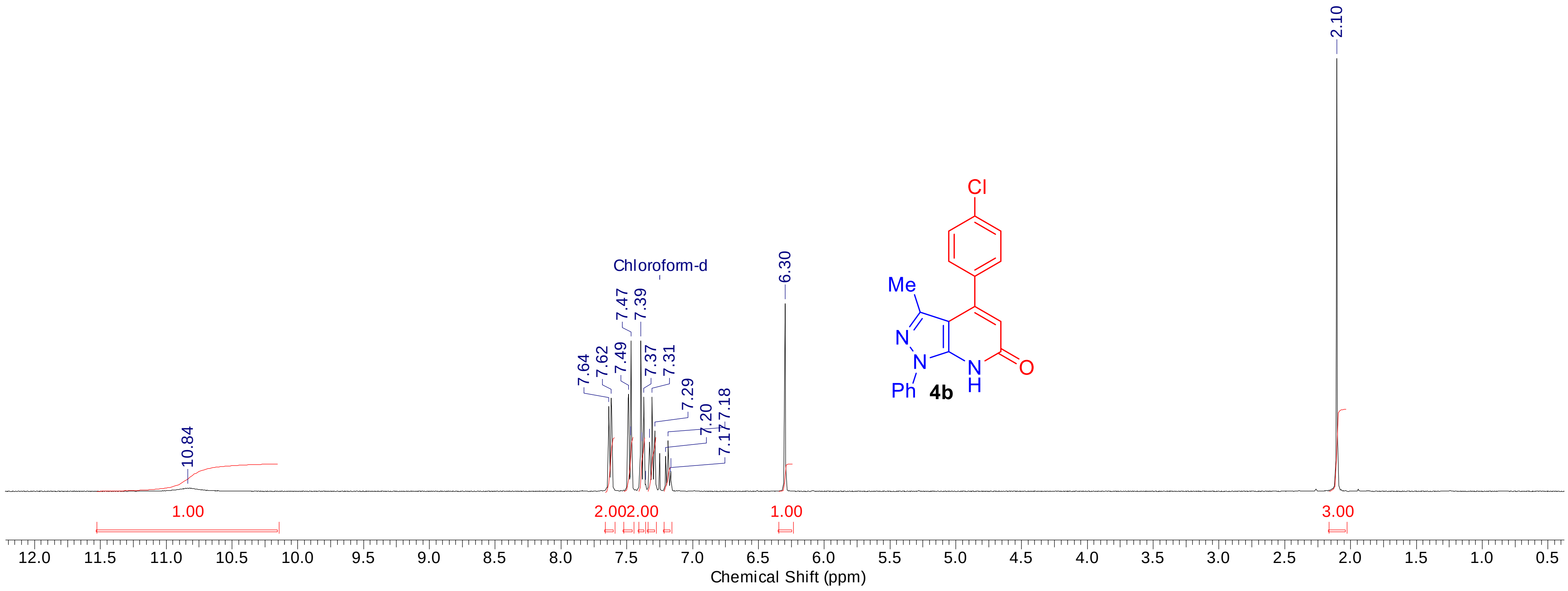

Figure S5: ^{1}H NMR spectrum of compound **4b** ($CDCl_3$).

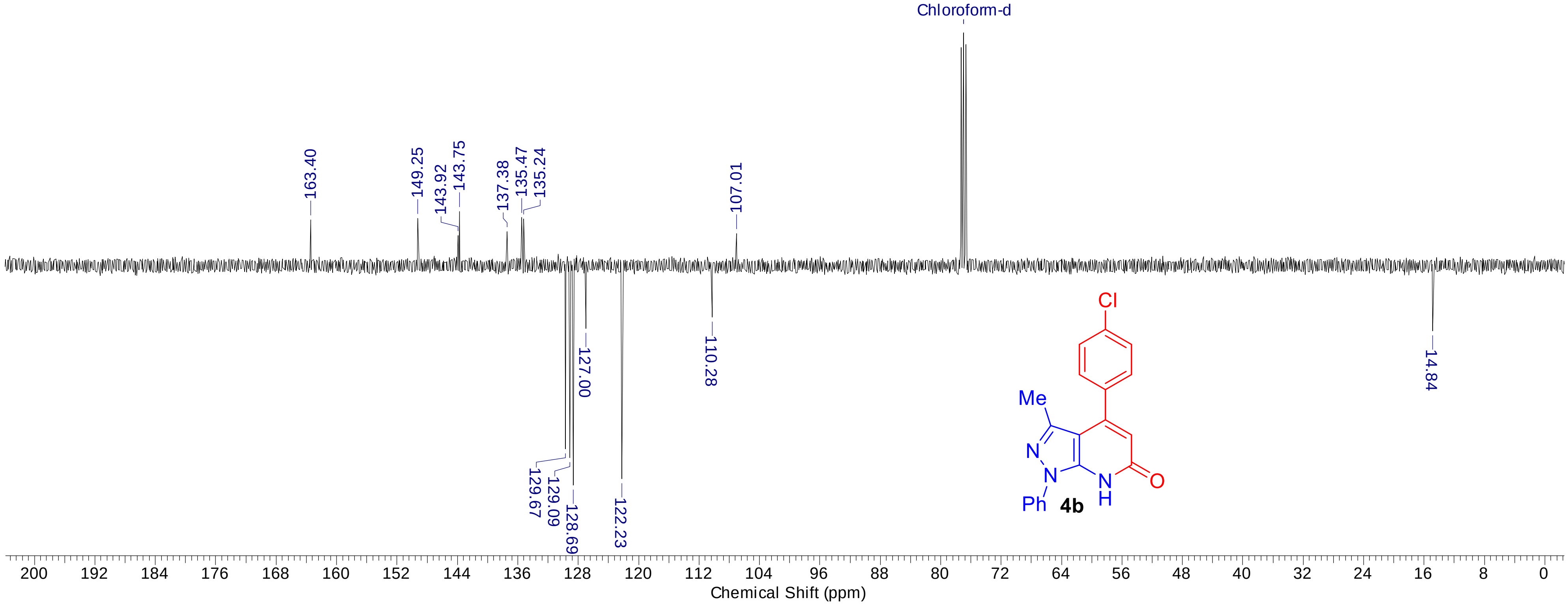

Figure S6: ^{13}C NMR spectrum of compound **4b** ($CDCl_3$).

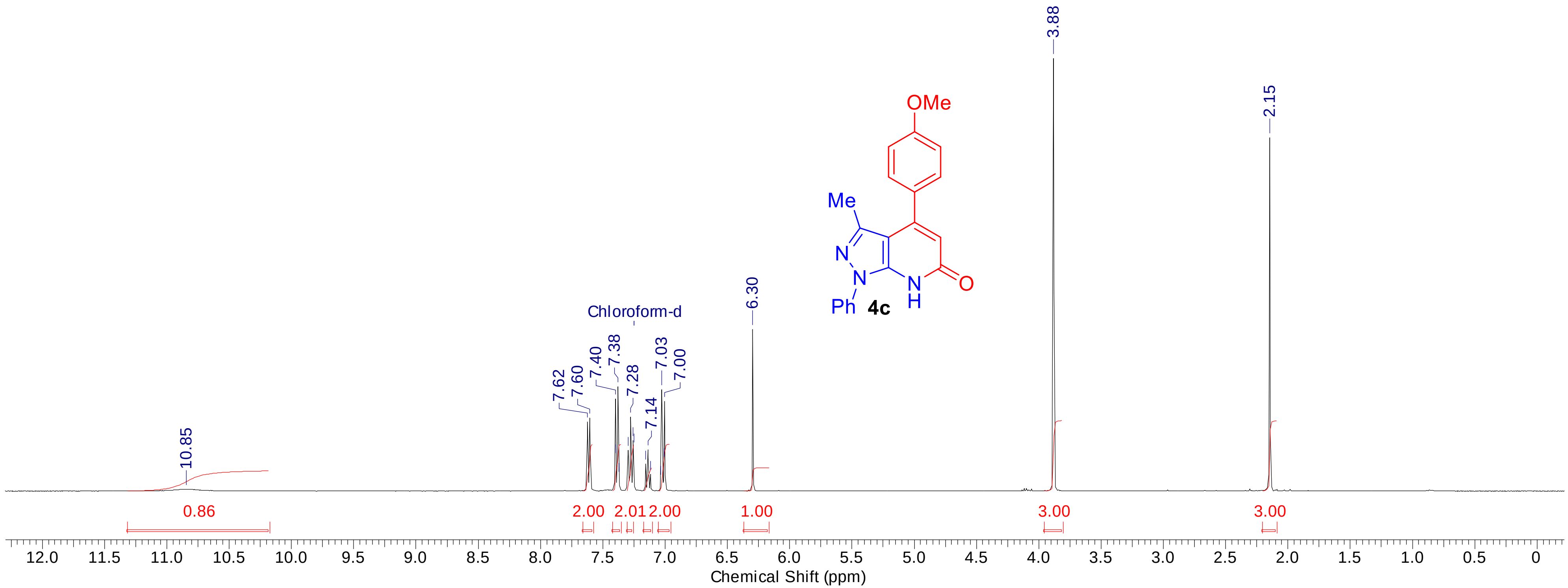

Figure S7: ^{1}H NMR spectrum of compound **4c** ($CDCl_3$).

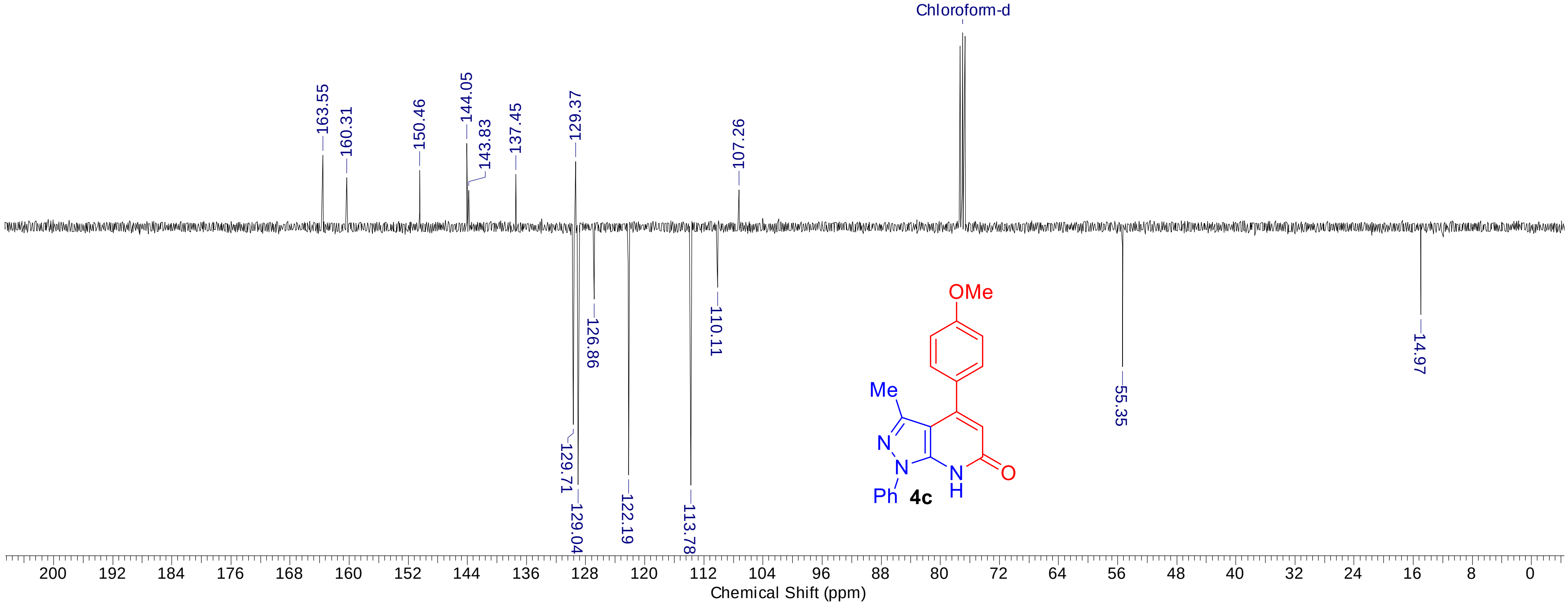

Figure S8: ^{13}C NMR spectrum of compound **4c** ($CDCl_3$).

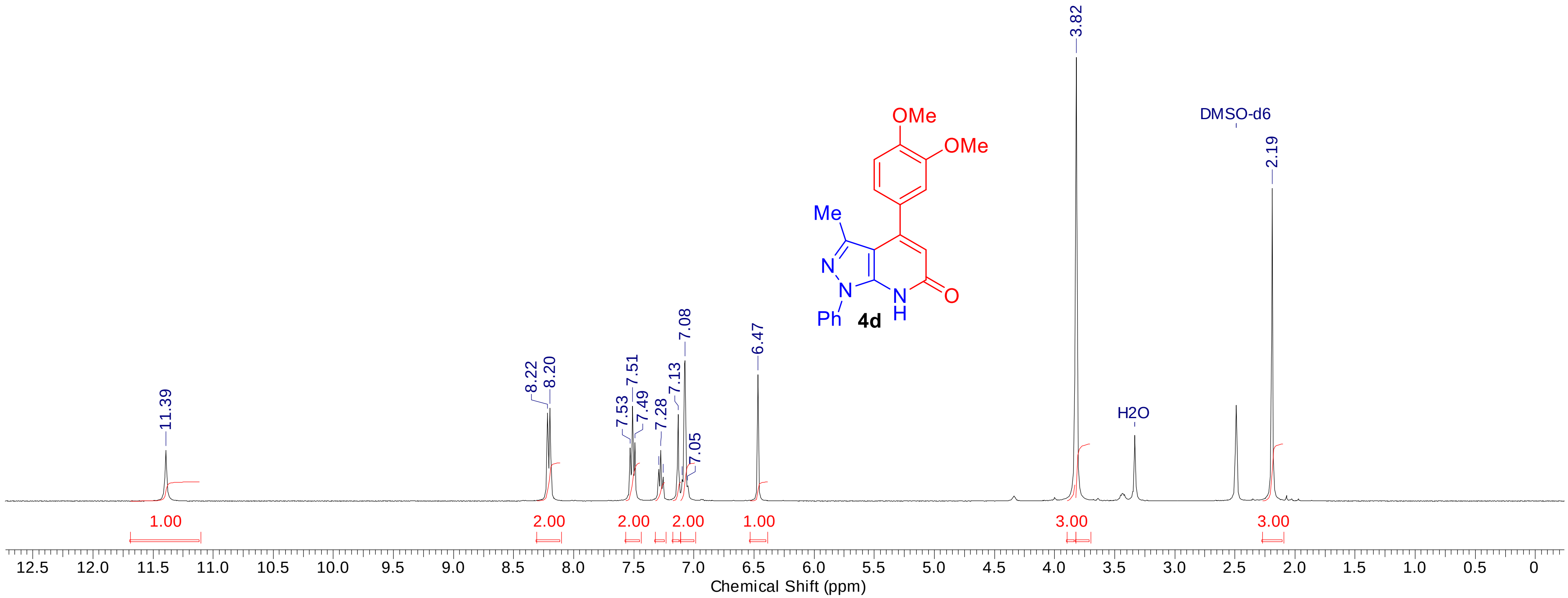

Figure S9: ^{1}H NMR spectrum of compound **4d** (DMSO-d_6).

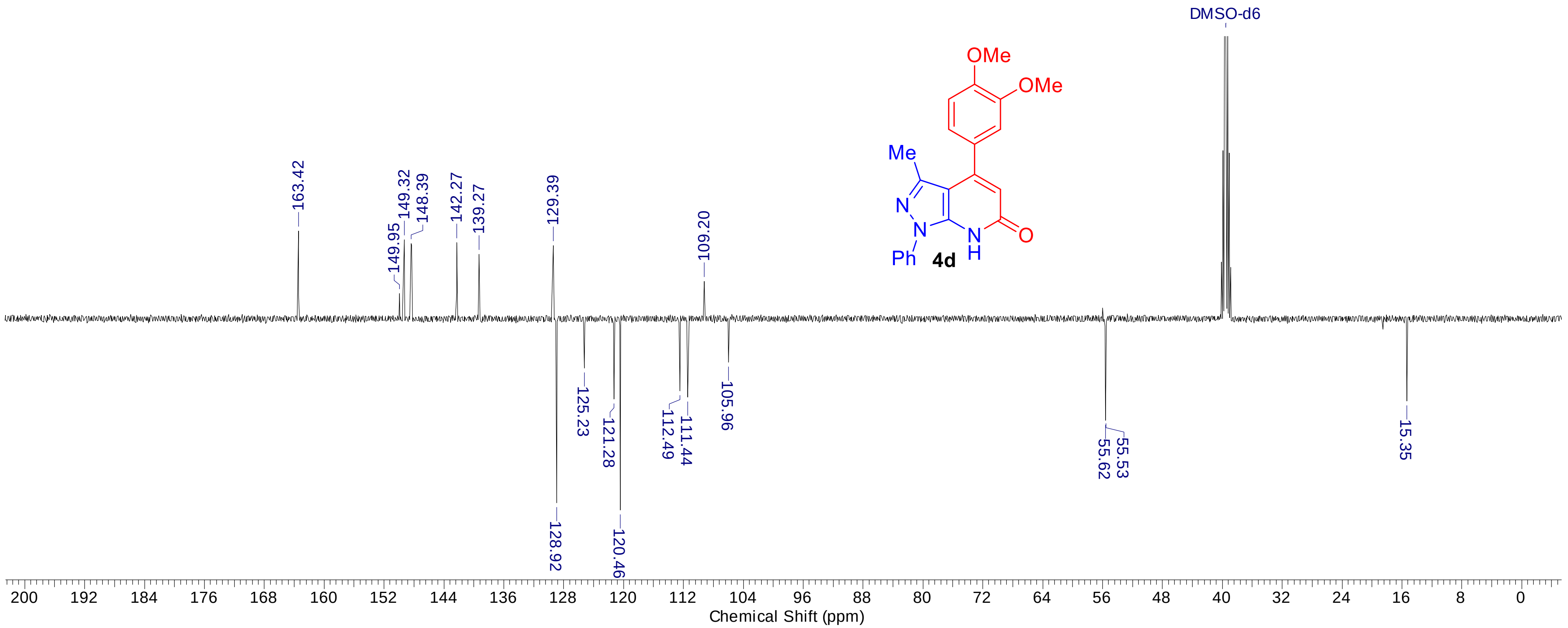

Figure S10: ^{13}C NMR spectrum of compound **4d** (DMSO-d_6).

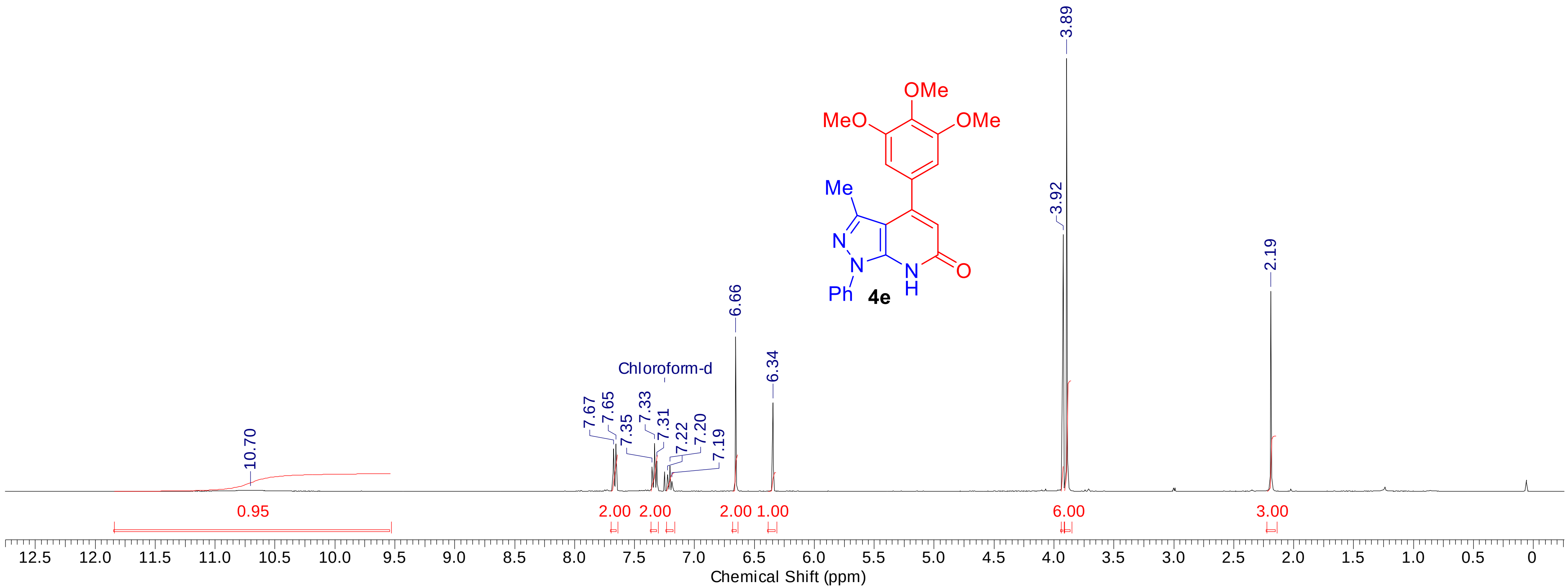

Figure S11: ^{1}H NMR spectrum of compound **4e** ($CDCl_3$).

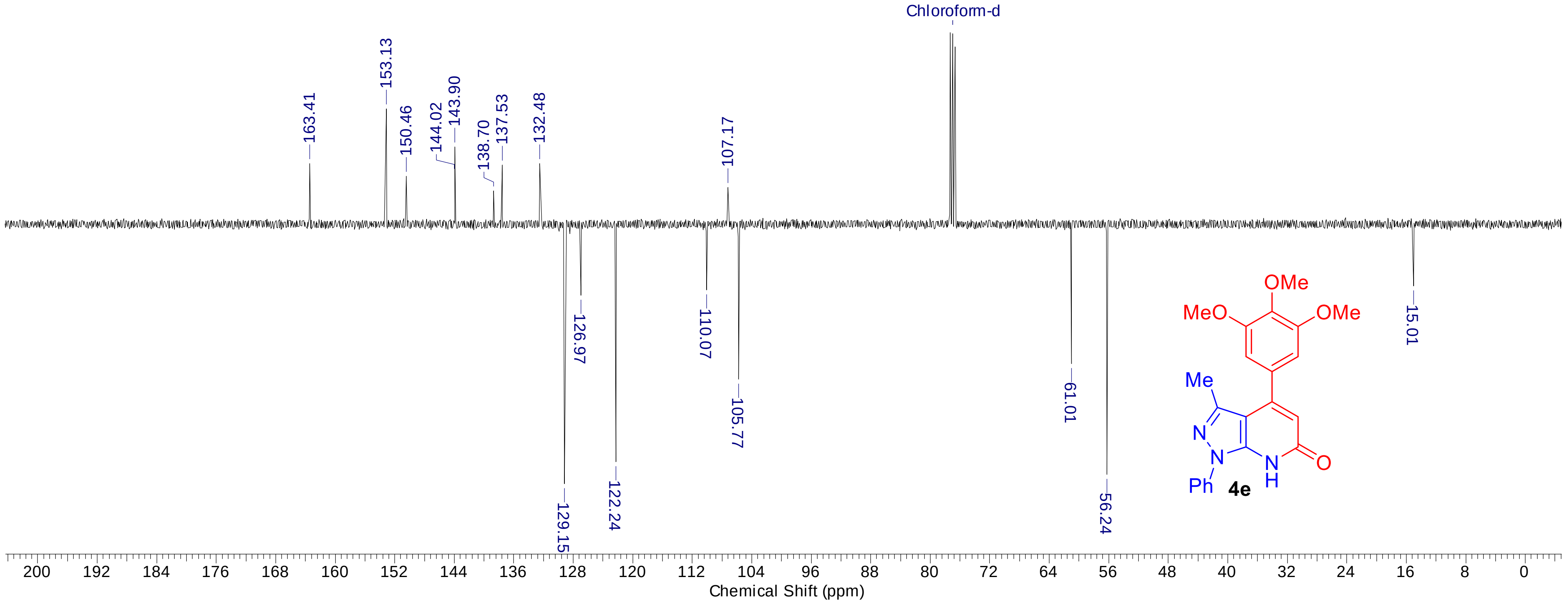

Figure S12: ^{13}C NMR spectrum of compound **4e** ($CDCl_3$).

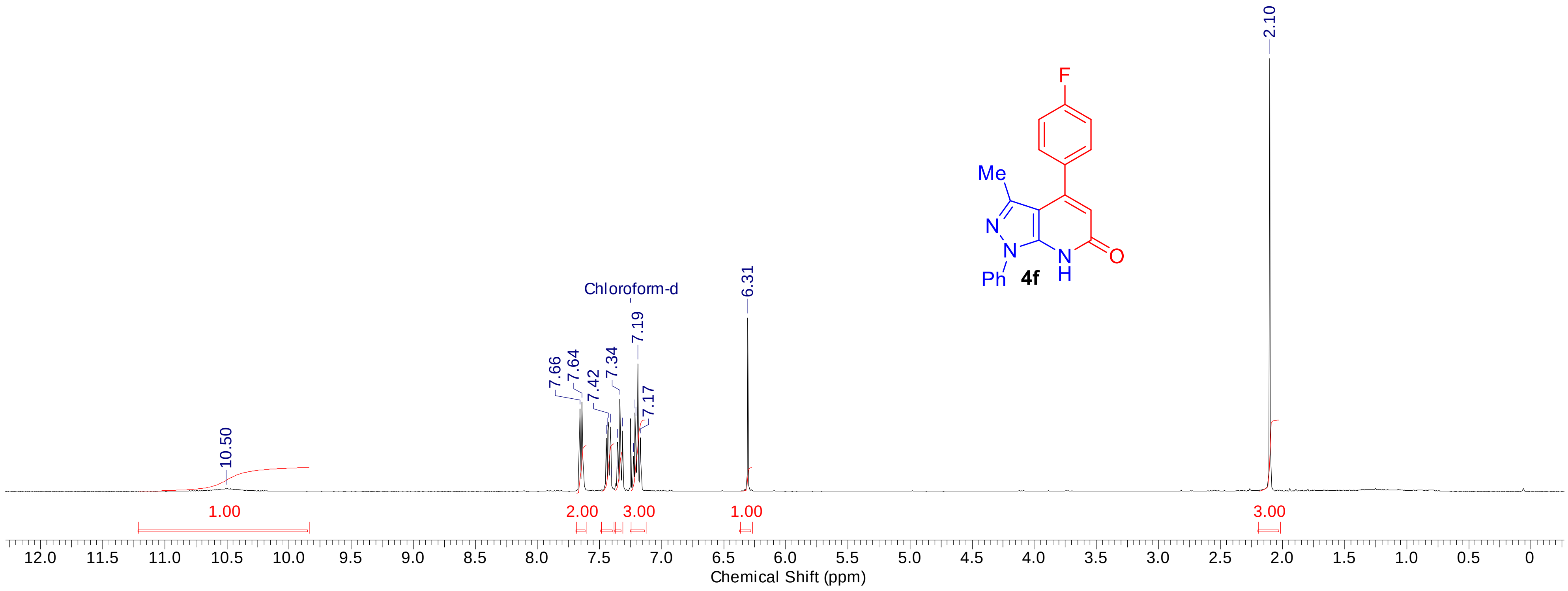

Figure S13: ^{1}H NMR spectrum of compound **4f** ($CDCl_3$).

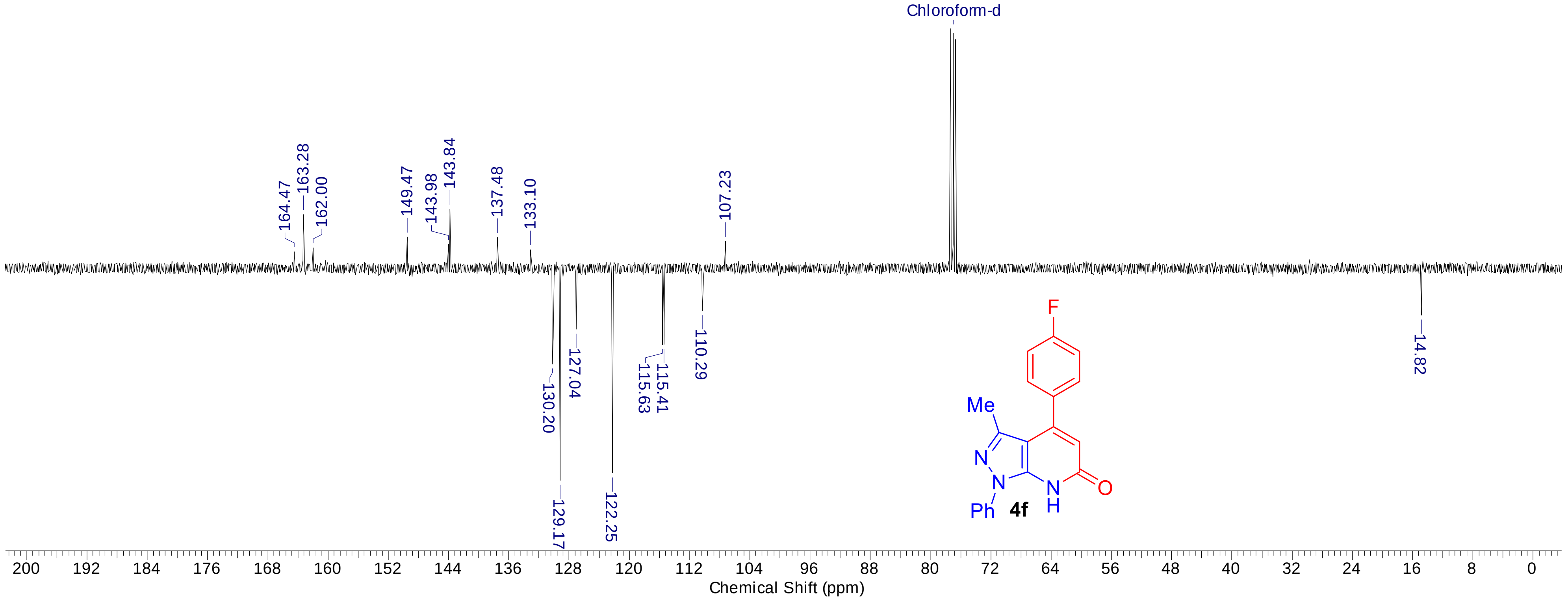

Figure S14: ^{13}C NMR spectrum of compound **4f** ($CDCl_3$).

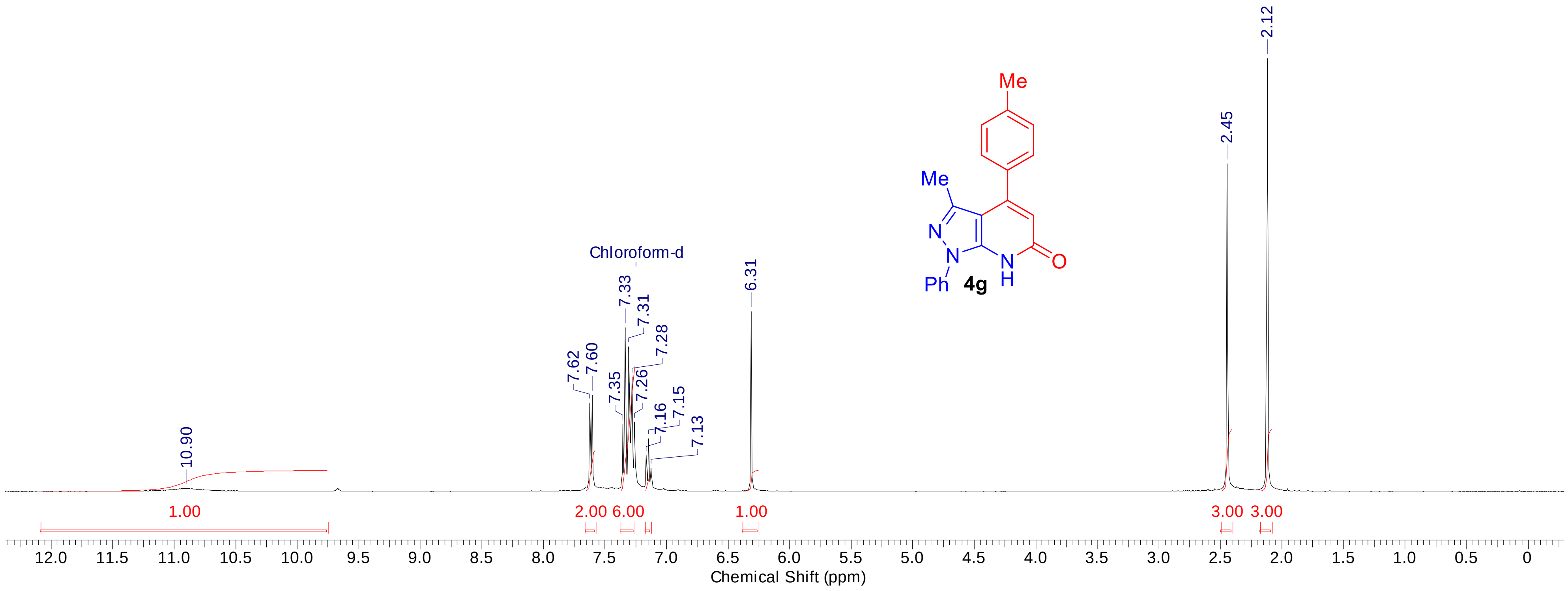

Figure S15: ^{1}H NMR spectrum of compound **4g** ($CDCl_3$).

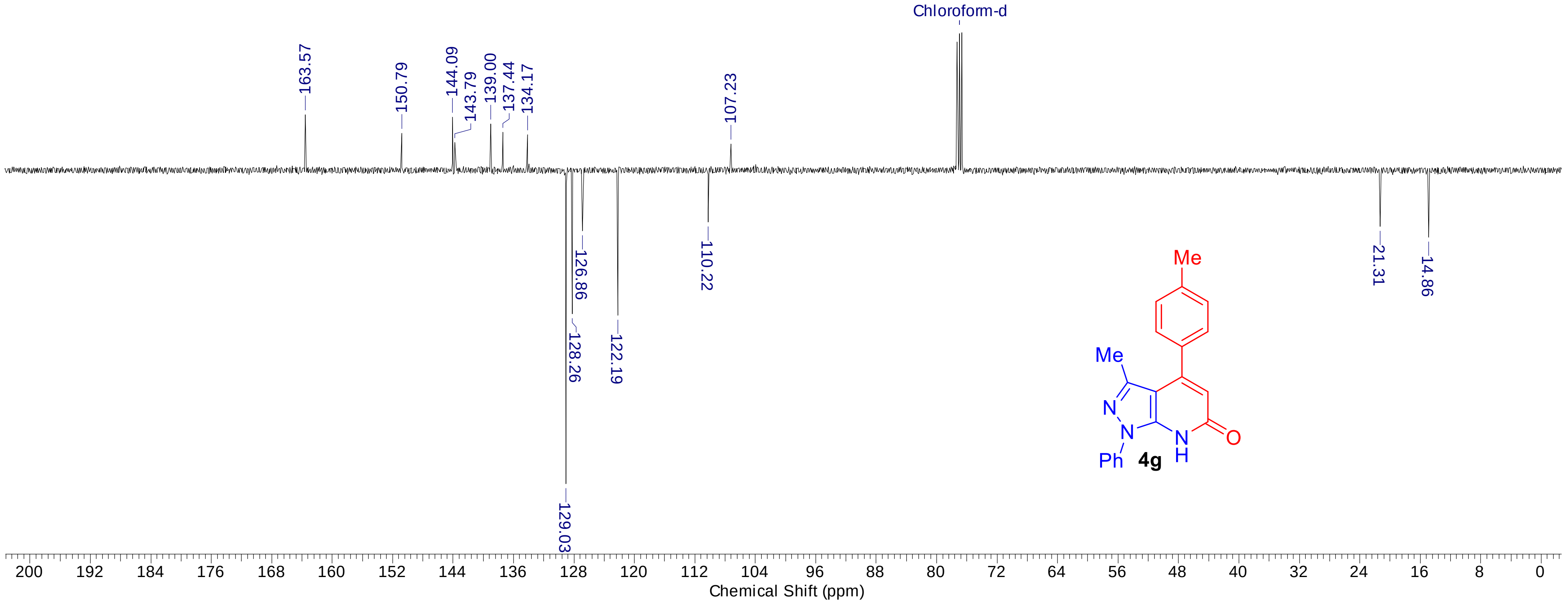

Figure S16: ^{13}C NMR spectrum of compound **4g** ($CDCl_3$).

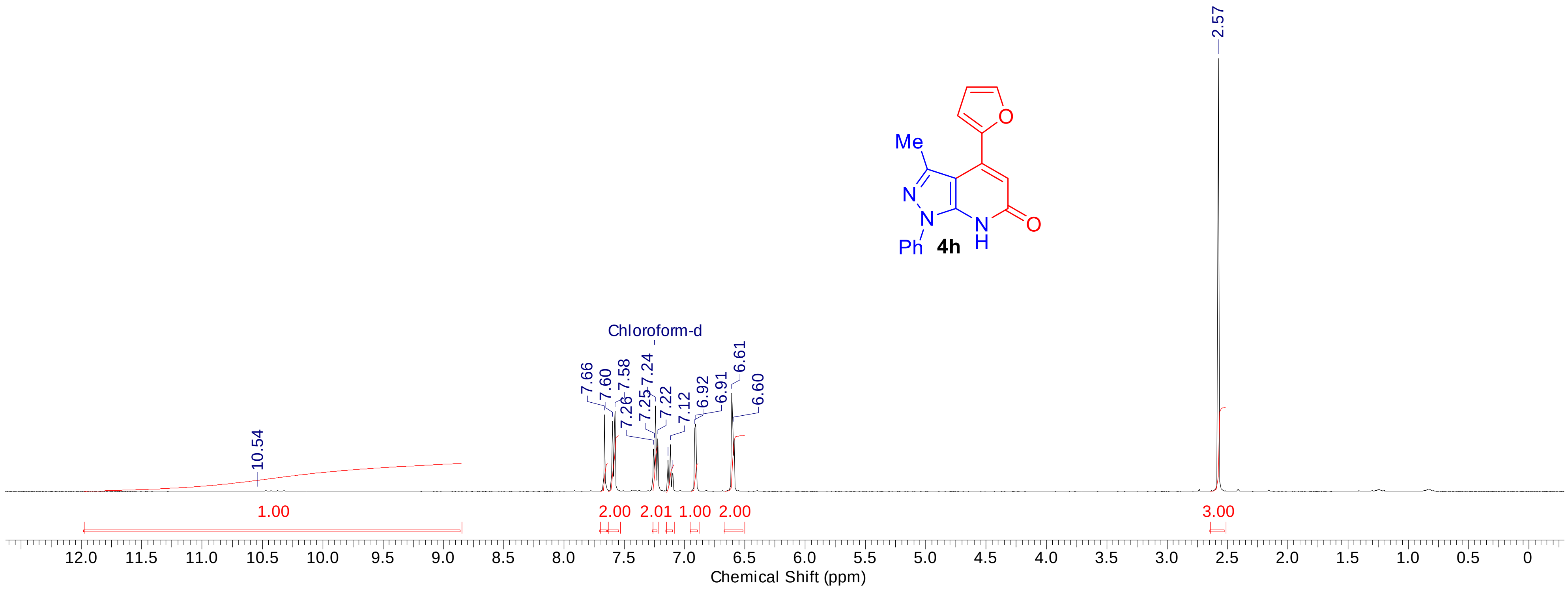

Figure S17: ^{1}H NMR spectrum of compound **4h** ($CDCl_3$).

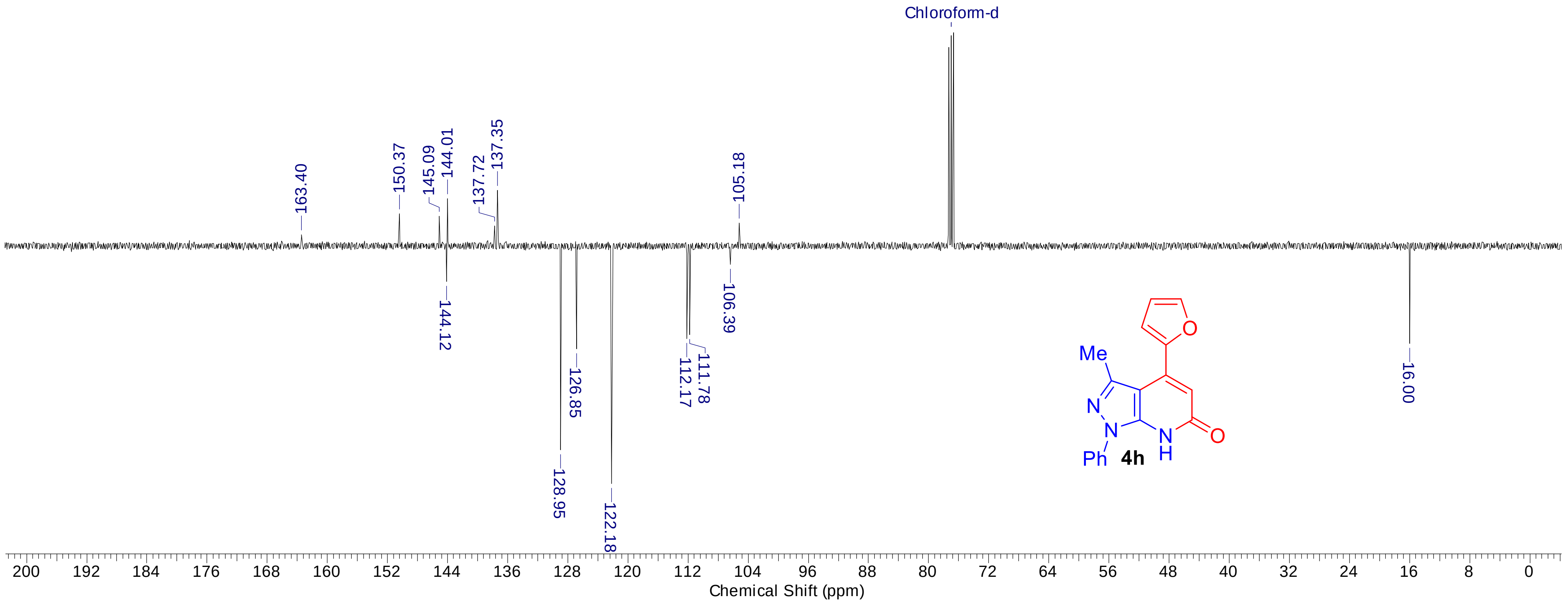

Figure S18: ^{13}C NMR spectrum of compound **4h** ($CDCl_3$).

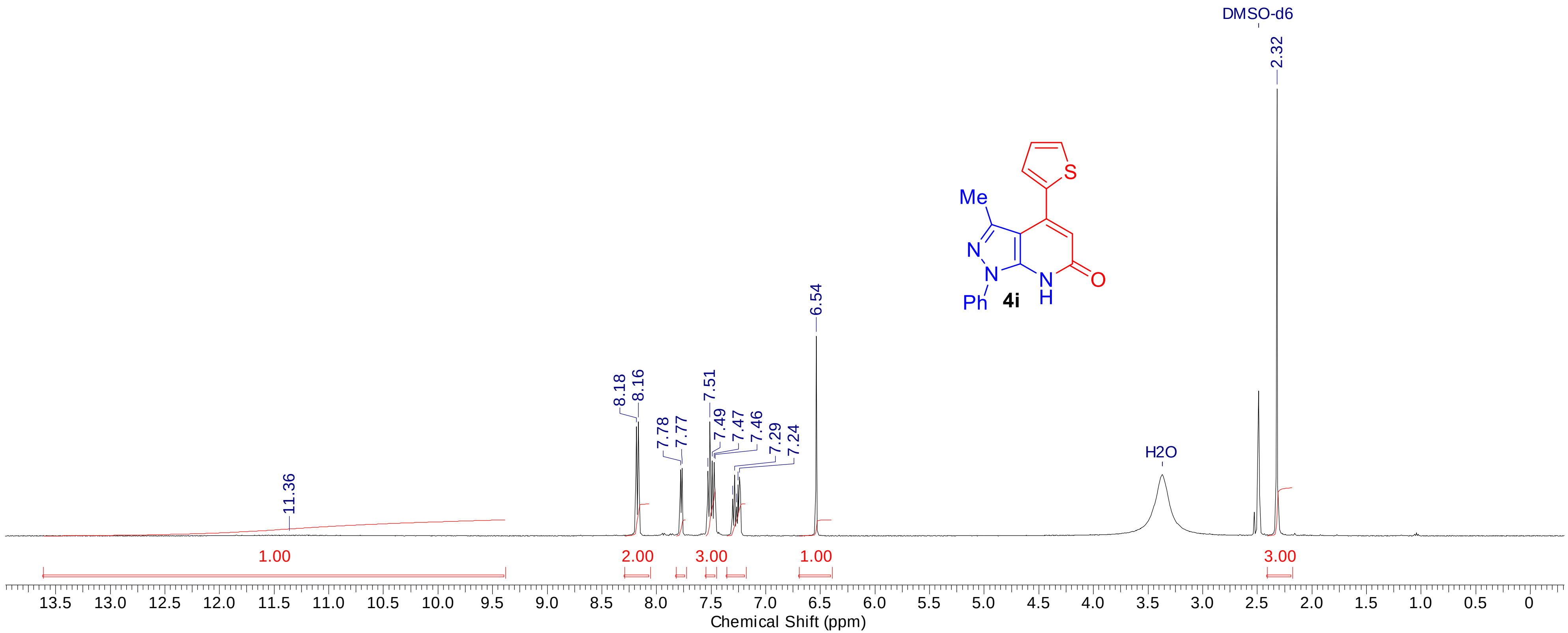

Figure S19: ^{13}C NMR spectrum of compound **4i** (DMSO-d_6).

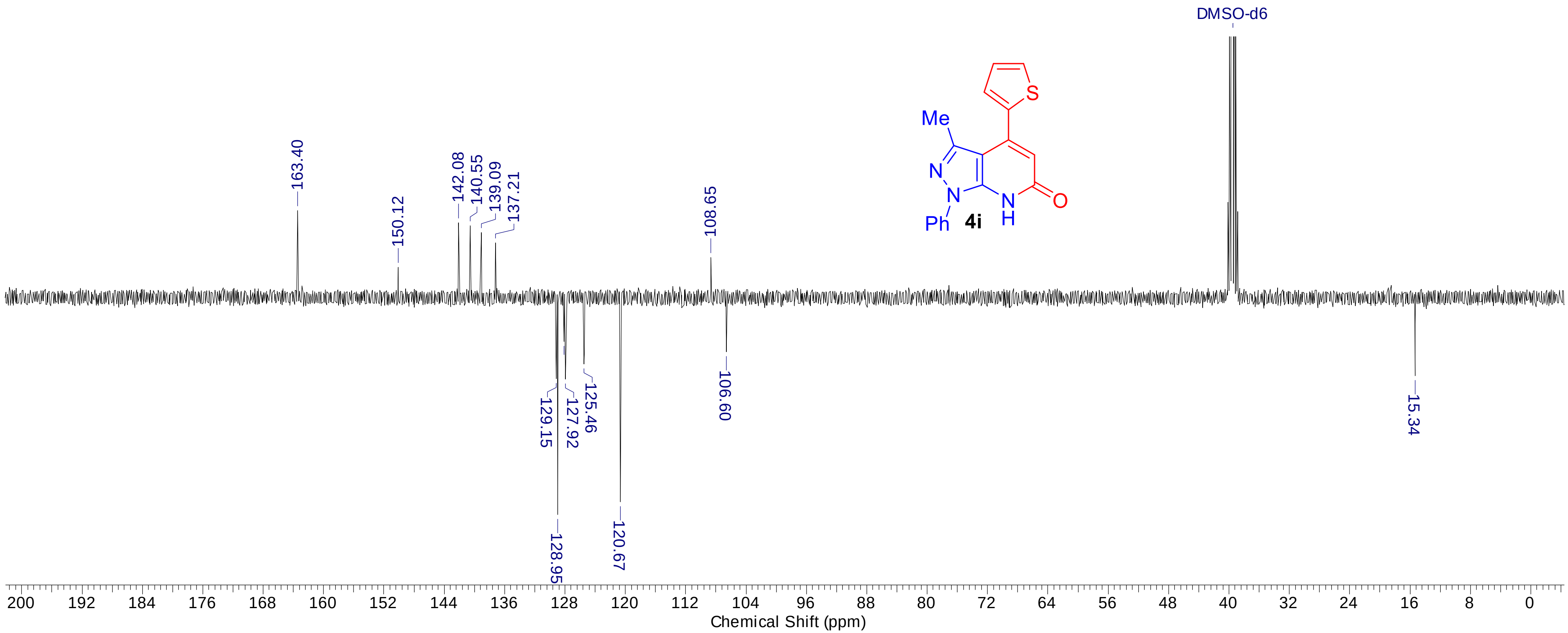

Figure S20: ^{13}C NMR spectrum of compound **4i** (DMSO-d_6).

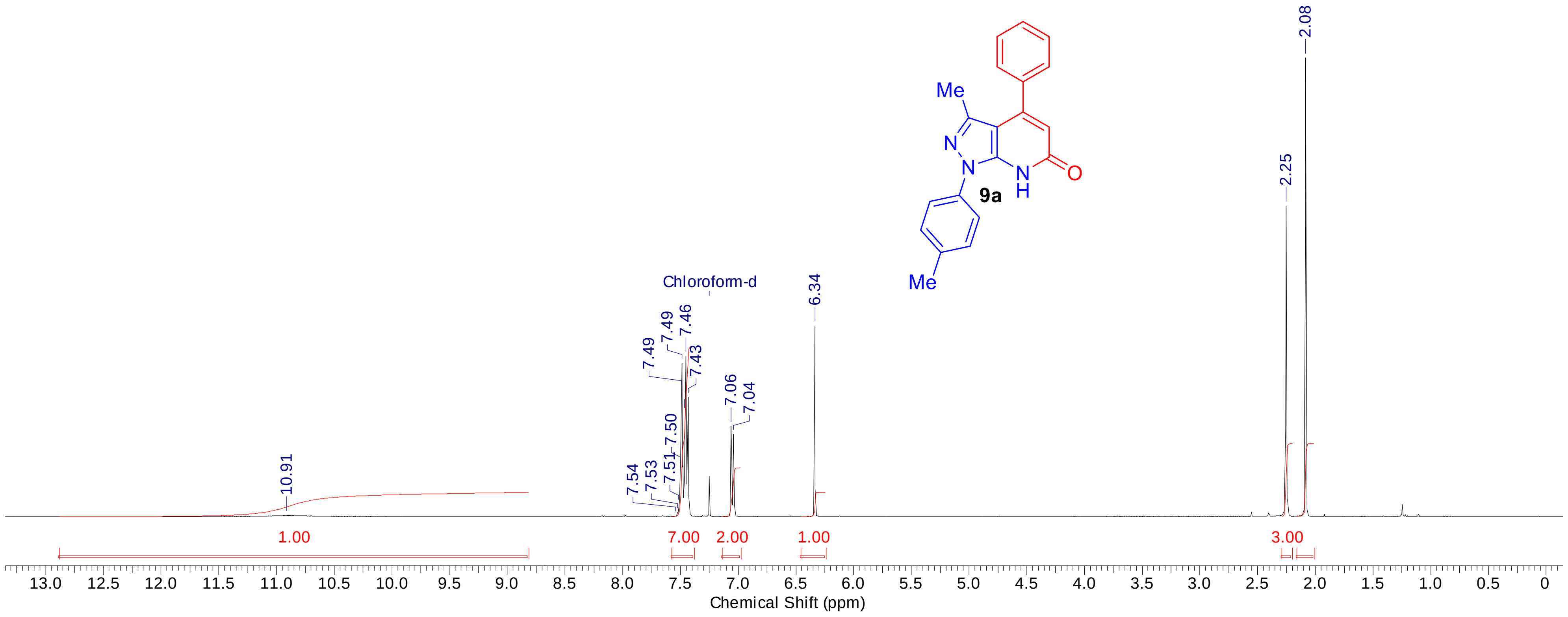

Figure S21: ^{1}H NMR spectrum of compound **9a** ($CDCl_3$).

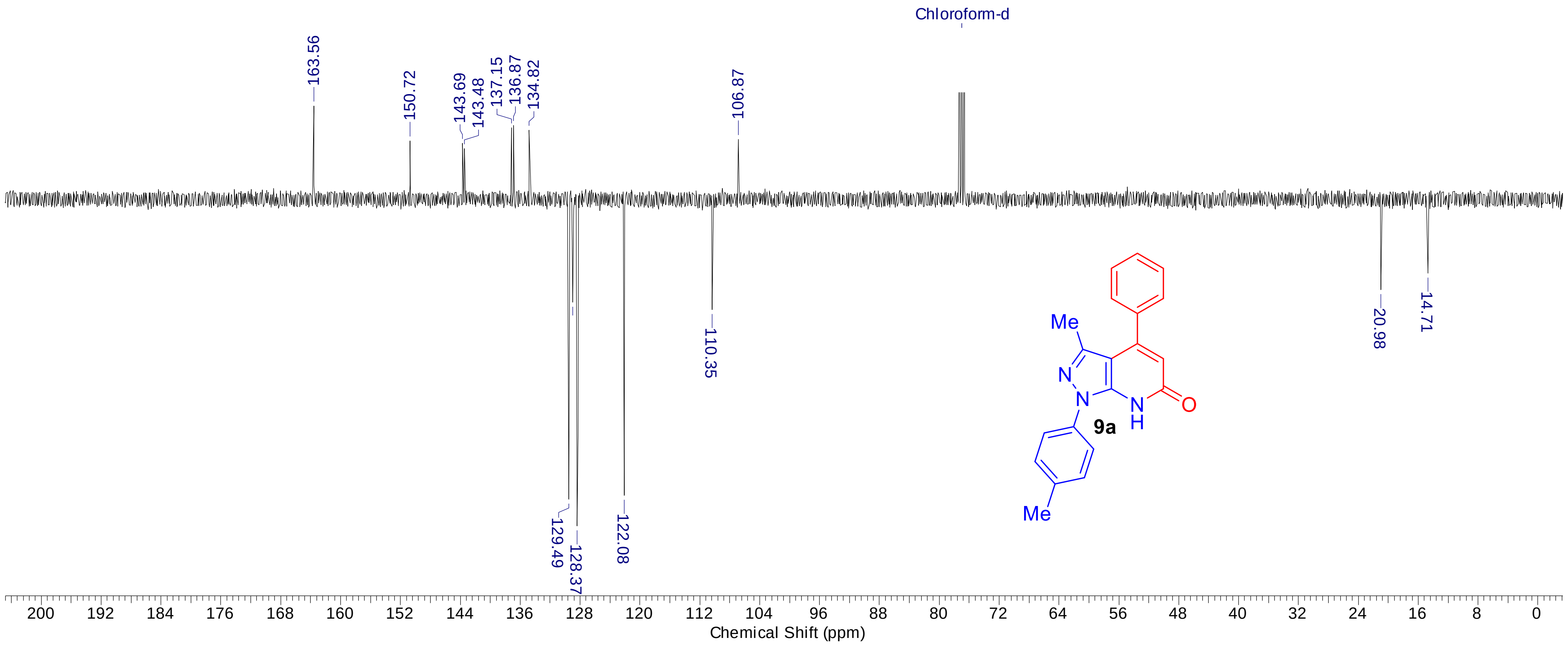

Figure S22: ^{13}C NMR spectrum of compound **9a** ($CDCl_3$).

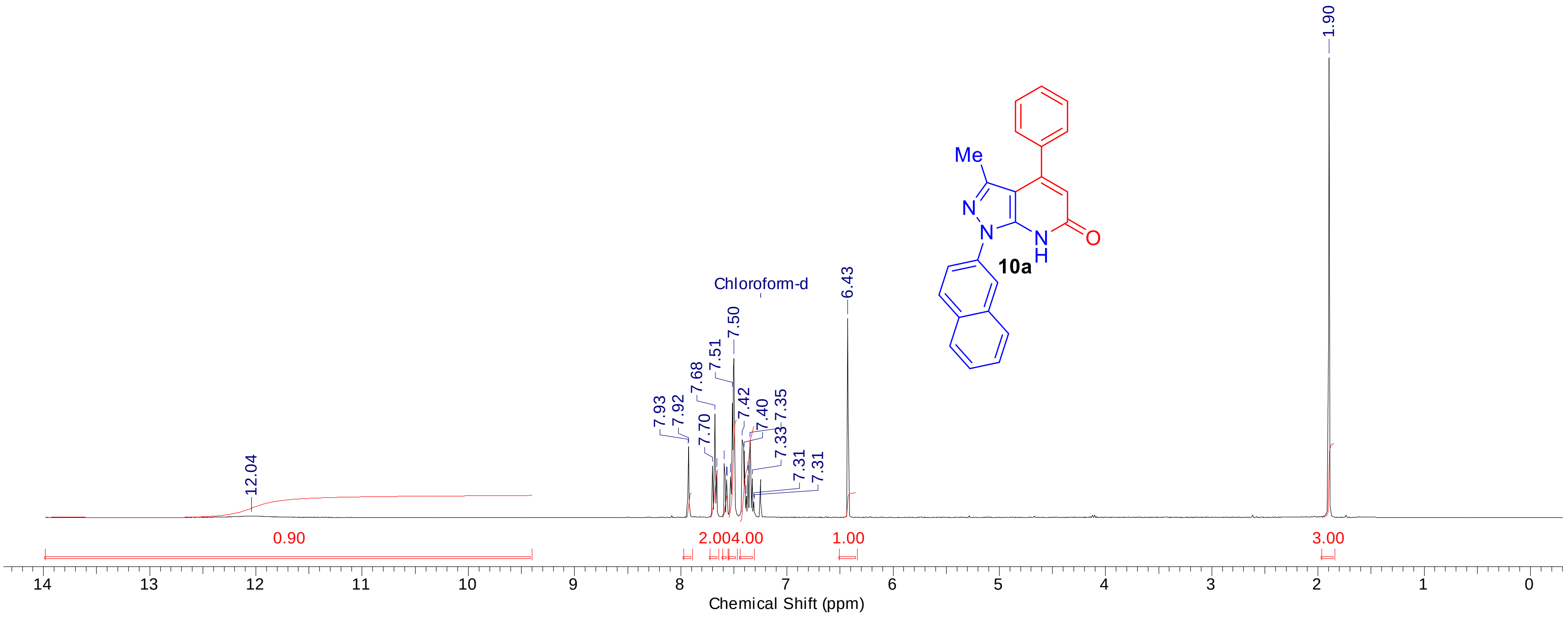

Figure S23: ^{1}H NMR spectrum of compound **10a** ($CDCl_3$).

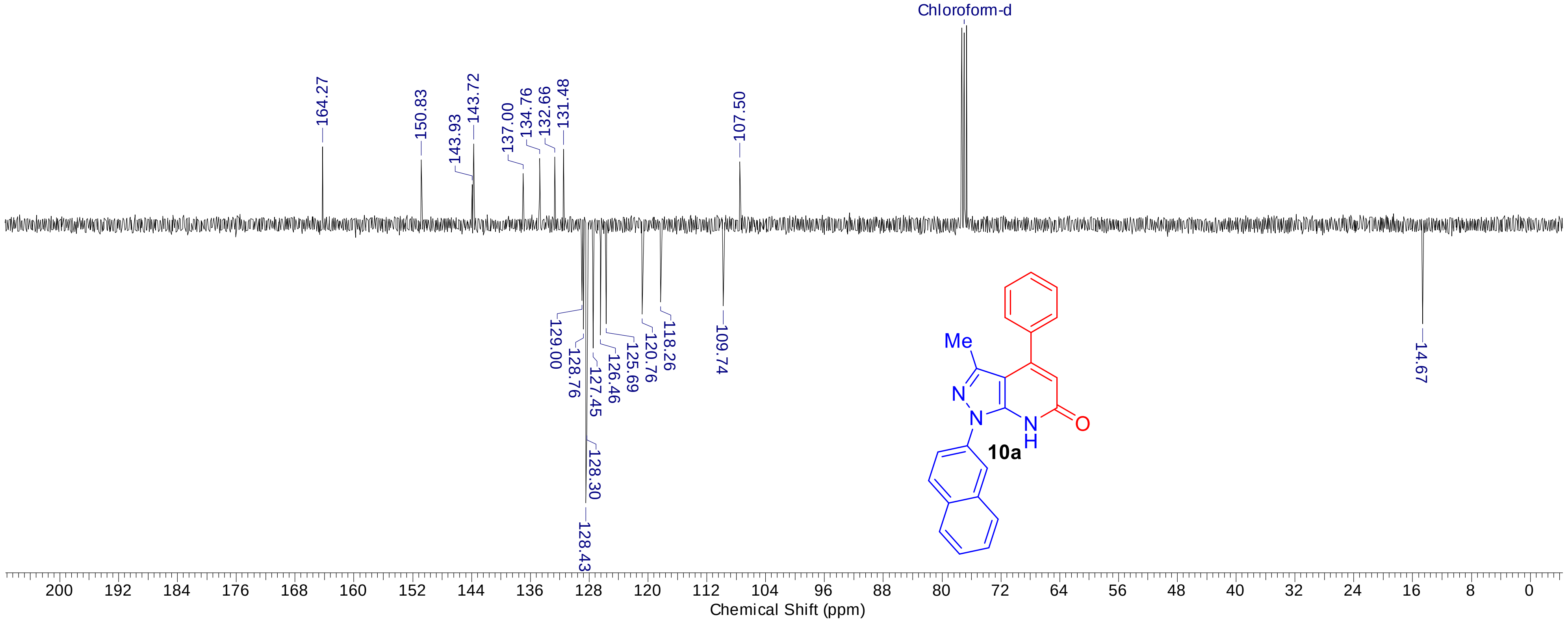

Figure S24: ^{13}C NMR spectrum of compound **10a** ($CDCl_3$).
